# Assessing the stability of free-energy perturbation calculations by performing variations in the method

**DOI:** 10.1007/s10822-018-0110-5

**Published:** 2018-03-13

**Authors:** Francesco Manzoni, Ulf Ryde

**Affiliations:** Theoretical Chemistry, Department of Chemistry, Chemical Centre, Lund University, P. O. Box 124, 221 00 Lund, Sweden

**Keywords:** Free-energy perturbation, Galectin-3, Independent simulations, Ligand-binding affinity, RESP charges, AM1-BCC charges

## Abstract

We have calculated relative binding affinities for eight tetrafluorophenyl-triazole-thiogalactoside inhibitors of galectin-3 with the alchemical free-energy perturbation approach. We obtain a mean absolute deviation from experimental estimates of only 2–3 kJ/mol and a correlation coefficient (*R*^2^) of 0.5–0.8 for seven relative affinities spanning a range of up to 11 kJ/mol. We also studied the effect of using different methods to calculate the charges of the inhibitor and different sizes of the perturbed group (the atoms that are described by soft-core potentials and are allowed to have differing coordinates). However, the various approaches gave rather similar results and it is not possible to point out one approach as consistently and significantly better than the others. Instead, we suggest that such small and reasonable variations in the computational method can be used to check how stable the calculated results are and to obtain a more accurate estimate of the uncertainty than if performing only one calculation with a single computational setup.

**Electronic supplementary material:**

The online version of this article (10.1007/s10822-018-0110-5) contains supplementary material, which is available to authorized users.

## Introduction

Predicting the affinity of a small molecule to a biomacromolecule is one of the greatest challenges in computational chemistry [1, 2]. If such binding affinities could be accurately predicted for arbitrary drug candidates, significant parts of drug development could be done by computers. Consequently, many methods have been developed for this aim [1], e.g. docking [3], MM/PBSA (molecular mechanics combined with Poisson–Boltzmann and solvent-accessible surface-area solvation) [4, 5] and linear interaction energy methods [6]. However, the most accurate results are typically obtained with free-energy perturbation (FEP) and other free-energy simulation methods [7, 8]. These are based on strict statistical mechanics theory and should in principle be limited only by the accuracy of the energy function employed and the sampling of the phase space.

Recent large-scale tests have shown that FEP calculations can often provide relative binding free energies with a mean absolute deviation (MAD) from experimental estimates of 4–6 kJ/mol [9–12]. However, the performance varies with the protein target and the type of ligands; in some cases, errors of over 20 kJ/mol are encountered. Likewise, the performance in prospective blind test (in which the experimental affinities are not know when the calculations are run), the performance is often worse, with root-mean-square-deviations (RMSD) of 6–18 kJ/mol [13, 14].

It has repeatedly been pointed out that the results of FEP calculations, as well as other binding-affinity calculations, strongly depend on the starting conditions [15–18]. Therefore, the uncertainty of a single calculation, even if it is quite long, gives a too optimistic estimate of the precision of the results. To obtain a valid error estimate, instead a number of statistically independent simulations should be run [15–22]. Such independent calculations are typically generated by employing different random-number seeds when setting up the starting velocities in the molecular dynamics (MD) simulations (these velocities are not known and therefore arbitrary, besides being taken from a Maxwell–Boltzmann distribution at the studied temperature). We have shown that the sampling can be further enhanced by employing other more or less arbitrary choices made during the setup of the simulations [20]. In particular, the solvation of the macromolecule in the simulation box is arbitrary, typically performed by overlaying an equilibrated box of solvent molecules and removing those that overlap with the solute. Therefore, equivalent independent simulations can be generated either by using different equilibrated solvent boxes or by simply rotating or translating the solute before the solvation [20]. This approach (together with different starting velocities), called solvent-induced independent trajectories, has been used in several of our studies [23–25].

However, there are many additional choices that the user needs to make during the setup of the simulation, e.g. selection of alternative conformation of disordered residues, the protonation of all residues with acid or base constants in the physiological range (in particular His residues), rotation of many groups, especially Asn, Gln, Ser, Thr and crystal-water molecules [20, 26]. These choices are of another type, because they are not completely arbitrary. Instead, there are typically better or worse choices and, in many cases, a single choice is correct. However, it may be very time-consuming to find the optimum choice in every case. Therefore, different users and software employ various heuristic approaches to make this assignment in a reasonable time, which naturally gives different solutions [27–31]. We have studied how these choices affect the results of the simulations and have shown that unless the affected site is close to the binding site of the ligand, the effect is minimal and therefore instead can be employed to enhance the sampling of the simulations [20].

In this paper, we examine another way to generate independent simulations. The user has to make additional choices when setting up the simulations, e.g. the force field, in particular the atomic partial charges, as well as the details of the FEP calculations. In the AMBER force fields [32], the partial charges are normally obtained by a restrained fit to the electrostatic potential (RESP) [33] around the molecule, calculated with quantum mechanical (QM) methods, typically at the Hartree–Fock/6–31G* level of theory. However, a cheaper method has also been developed, based on semiempirical calculations and bond charge corrections (AM1-BCC) [34]. The two sets of charges have been parametrised to give similar results [35, 36]. In addition, it is not fully specified how the molecule of interest should be treated before the charges are calculated. Often, it is optimised to give bonds and angles compatible with the QM method, but for elongated polar molecules, the molecule may curl up to make as many internal hydrogen bonds as possible. In this paper, we compare results obtained with RESP and AM1-BCC charges and optimised with either with Hartree–Fock or AM1. Moreover, we compare two selections of the perturbed group, viz. either only the atoms that differ between the two ligands or the whole chemical group involved (a tetrafluorophenyl ring). The perturbed group affects both which atoms are assigned soft-core potentials and which atoms are allowed to have differing coordinates [37]. We first show that the six different FEP schemes employed give nearly identical results, without any clear trends indicating that any of the approaches is better than the others, and therefore can be combined to provide enhanced sampling by variations in the computational method.

As the test case, we employ the binding of eight substituted tetrafluorophenyl-triazole-thiogalactosides to the carbohydrate-recognition domain of galectin-3 [38]. Galectin-3 is a mammalian β-galactoside binding protein involved in glycoprotein trafficking, signalling, cell adhesion, angiogenesis, macrophage activation and apoptosis [39–43]. It has been implicated in inflammation, immunity, cancer development and metastasis [44]. Previous FEP studies of this protein have given quite large errors in the calculated relative binding affinities [45], but in this study, all calculations give excellent results, with MADs of 2–3 kJ/mol.

## Computational methods

### Molecular dynamics simulations

The molecular dynamics (MD) and FEP calculations were based on the X-ray crystal structure of a related compound bound to galectin-3C (PDB id 5E89) [46]. The various ligands were built by manually modifying this ligand. All crystal-water molecules were kept in the simulations. Each galectin-3 complex was solvated in an octahedral box of water molecules extending at least 10 Å from the solute using the tleap module of the Amber software [32], so that 4965–5593 water molecules were included in the simulations. The simulations were set up in the same way as in our previous studies of galectin-3 [26, 31, 47]: All Glu and Asp residues were assumed to be negatively charged and all Lys and Arg residues positively charged, whereas the other residues were neutral. His-158 and 217 were protonated on the ND1 atoms, whereas and the other two His residues were protonated on the NE2 atom, in accordance with NMR measurements and previous extensive test calculations with MD [31]. This gave a net charge of + 4 for the protein. No counter ions were used in the simulations. The protein was described by the Amber 99SB force field [48], water molecules with the TIP3P force field [49], whereas the ligands were treated with the general Amber force field [50].

Three different sets of charges were employed for the ligands: They were obtained either with the RESP method [33], using electrostatic potentials calculated with the Hartree–Fock/6–31G* approach or with the cheaper AM1-BCC approach [34, 35]. In the former case, geometries were first optimised at the Hartree–Fock/6–31G level of theory, giving rise to the RH charge set. In the latter case, we either used the same geometries (BH charges) or geometries optimised with the semiempirical AM1 method (BA charges) [51]. The various charges are shown in Table S2 in the supplementary material. The BH and BA charges are quite similar with a mean absolute deviation (MAD) of 0.007 e, whereas the RH charges are more different with a MAD of 0.11 e to the other two charge sets.

The MD simulations were performed with the sander module of Amber 14 [32]. The temperature was kept constant at 300 K using a Langevin thermostat [52] with a collision frequency of 2.0 ps^−1^, and the pressure was kept constant at 1 atm using a weak-coupling isotropic algorithm [53] with a relaxation time of 1 ps. Particle-mesh Ewald summation [54] with a fourth-order B spline interpolation and a tolerance of 10^−5^ was used to calculate electrostatic energies and forces. The cut-off for the Lennard–Jones interactions was set to 8 Å and the non-bonded pair list was updated every 50 fs. The SHAKE algorithm [55] was used to constrain bonds involving hydrogen atoms so that a 2 fs time step could be used.

### Free-energy perturbations

**Fig. 1:**
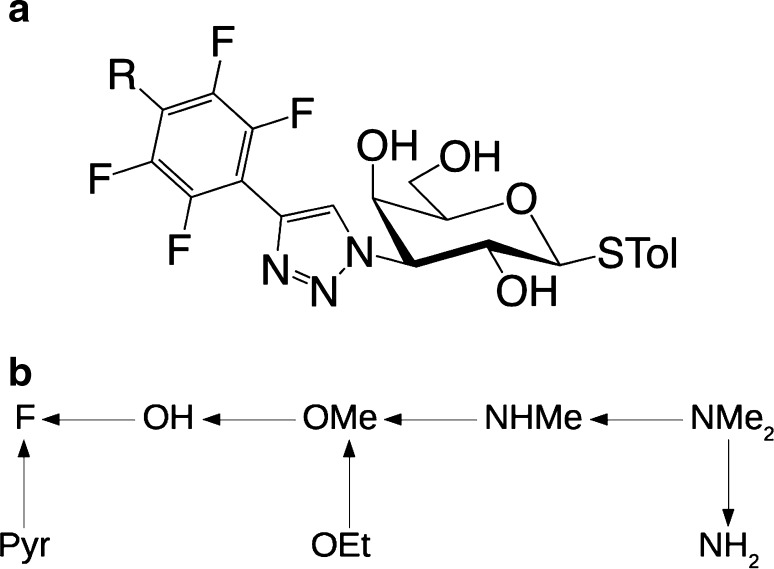
**a** The eight studied galectin-3 inhibitors with R = F, OH, OMe, OEt, NH_2_, NHMe, NMe_2_, or pyrrolidine. STol is *S*-para-toluene. **b** The seven transformations studied by FEP

We have calculated the relative free energy of the eight inhibitors for galectin-3 in Fig. 1. They are all 3-(4-(2,3,5,6-tetrafluorophenly)-1,2,3-triazol-1-yl)-thiogalactosides with a varying substituent in the para-position on the tetrafluorophenyl group. They will simply be named after this varying substituent, F, OH, OMe, OEt, NH_2_, NHMe, NMe_2_, or pyrrolidine (Pyr). These inhibitors were connected by seven transformations, keeping the chemical differences as small as possible: OH → F, OMe → OH, OEt → OMe, NHMe → OMe, NMe_2_ → NHMe, NMe_2_ → NH_2_ and Pyr → F, as is shown in Fig. 1b.

Relative binding free energies (∆∆*G*_bind_) were calculated for these transformations with FEP using a thermodynamic cycle that involves the conversion of one ligand (L_1_) to the other (L_2_) both in the protein binding site and in solution [25, 56]. The free energies of the transformations were calculated using the multi-state Bennett acceptance ratio (MBAR) approach, calculated with the PYMBAR software [57]. We employed the single transformation-approach [25, 58] in which both electrostatic and van der Waals interactions are modified in the same step. The calculations employed soft-core versions of both the van der Waals and Coulomb potentials [59, 60].

To improve the convergence of the free-energy difference, the transformation L_1_ → L_2_ was divided into several small steps, involving intermediate states, defined by the potential energy *V*(λ) = (1 − λ) *V*_0_ + λ*V*_1_, where *V*_0_ and *V*_1_ are the potential energies of the L_1_ and L_2_ states, respectively. Thirteen λ values were used (0, 0.05, 0.1, 0.2, 0.3, 0.4, 0.5, 0.6, 0.7, 0.8, 0.9, 0.95 and 1). In all calculations, we used a single-topology scheme and the pmemd module of the Amber software with two sets of coordinates for the atoms that differ between L_1_ and L_2_ [37].

In the FEP calculations, we included in the perturbed group either only atoms directly involved in the perturbation (SP, small perturbed group), i.e. those in the para substituent, or all atoms of the terminal substituted tetrafluorophenyl group (LP, large perturbed group). In the present implementation of FEP [37], the perturbed group not only defines what atoms have a varying force field (including the soft-core Lennard–Jones and Coulomb potential), but also the atoms that are allowed to have different coordinates in the simulations.

The FEP simulations were performed in the following way: The system at each λ value was minimized for 500 cycles, with all atoms except water molecules and hydrogen atoms restrained to their start position with a force constant of 418 kJ/mol/Å^2^. This was followed by a 20 ps constant-pressure simulation, using the same constraints, and a 1 ns constant-pressure simulation without any restraints. Finally, a 5 ns constant-pressure production run was performed, during which coordinates and energies were sampled every 10 ps. In total, six sets of calculations were performed, which will be denoted by the charges employed for the ligands (RH, BA or BH) and the size of the perturbed group (SP or LP), e.g. BA/LP.

### Error estimates, quality and overlap measures

All reported uncertainties are standard errors of the mean (standard deviations divided by the square root of the number of samples). The uncertainty of the MBAR free energies calculated at each *λ* value was estimated by bootstrapping using the PYMBAR software [57] and the total uncertainty was obtained by error propagation.

The performance of the free-energy estimates was quantified by the mean absolute deviation (MAD), the mean signed deviation (MSD), the root-mean-square deviation (RMSD), the maximum error (Max), the correlation coefficient (*R*^2^) and Kendall’s rank correlation coefficient (τ_r_) compared to experimental data [38]. The latter was calculated only for the transformations that were explicitly studied, not for all combinations that can be formed from these transformations. Moreover, it was also evaluated considering only differences (both experimental and calculated) that are statistically significant at the 90% level (τ_r, 90_) [37]. Note that *R*^2^ depends on the direction of the perturbation (i.e. whether L_1_ → L_2_ or L_2_ → L_1_ is considered, which is arbitrary). This was solved by considering both directions (both forward and backward) for all perturbations when these two measures were calculated. The standard deviation of the quality measures was obtained by a simple simulation approach [17]. For each transformation, 1000 Gaussian-distributed random numbers were generated with the mean and standard deviation equal to the MBAR and experimental results for that transformation. Then, the quality measures were calculated for each of these 1000 sets of simulated results and the standard error over the 1000 sets is reported as the uncertainty.

For all *λ* values of all perturbations, we have monitored five overlap measures, to ensure that the overlap of the studied distributions is satisfactory, viz. the Bhattacharyya coefficient Ω [61], the Wu and Kofke overlap measures of the energy probability distributions (*K*_AB_) [62] and their bias metrics (Π) [62], the weight of the maximum term in the exponential average (*w*_max_) [63] and the difference of the forward and backward exponential average estimate (ΔΔ*G*_EA_) [10]. In all calculations, Π > 0.4 and the other overlap measures were also in the safe range [10, 64], except in a single case for the OMe → OH perturbation with BH/LP (*w*_max_ = 0.7). Therefore, we conclude that the overlap is satisfactory for the simulations.

## Result and discussion

We have studied the binding affinity of the eight substituted tetrafluorophenyl-triazole-thiogalactoside inhibitors of galectin-3, shown in Fig. 1a. Relative binding free energies were calculated for seven pairs of ligands, as is illustrated in Fig. 1b. The affinities were calculated by FEP with the MBAR approach. They are compared to experimental affinities obtained by competitive fluorescence polarization measurements (Table 1) [38, 65, 66].

**Table 1:** Calculated relative binding free energies (kJ/mol), obtained with three different sets of charges for the ligands (RH, BA and BH) and two perturbed groups (SP or LP)

	RH/SP	RH/LP	BA/SP	BA/LP	BH/SP	BH/LP	Consensus	Exp.
OMe → OH	0.2 ± 0.4	1.0 ± 0.7	− 0.8 ± 0.4	1.6 ± 0.6	− 0.6 ± 0.4	0.9 ± 0.6	0.4 ± 0.4	0.6 ± 0.3
NHMe → OMe	− 0.8 ± 0.3	− 3.3 ± 0.6	− 6.0 ± 0.4	− 4.8 ± 0.6	− 6.7 ± 0.4	− 6.4 ± 0.6	− 4.7 ± 0.9	0.0 ± 0.3
NMe_2_ → NHMe	− 5.8 ± 0.5	− 4.4 ± 0.7	− 1.6 ± 0.5	− 1.1 ± 0.6	− 3.2 ± 0.5	− 2.2 ± 0.7	− 3.0 ± 0.7	− 2.0 ± 0.2
NMe_2_ → NH_2_	− 1.7 ± 0.5	− 2.9 ± 0.7	− 3.9 ± 0.5	− 3.6 ± 0.7	− 2.9 ± 0.5	− 5.1 ± 0.7	− 3.3 ± 0.5	− 3.2 ± 0.2
OEt → OMe	− 2.8 ± 0.4	2.7 ± 0.7	− 4.2 ± 0.4	− 1.4 ± 0.6	− 3.3 ± 0.4	− 2.7 ± 0.6	− 1.9 ± 1.0	− 4.0 ± 0.4
Pyr → F	− 10.4 ± 0.6	− 7.5 ± 0.8	− 10.4 ± 0.6	− 9.1 ± 0.7	− 9.0 ± 0.6	− 8.3 ± 0.7	− 9.1 ± 0.5	− 11.2
OH → F	− 0.4 ± 0.2	− 2.1 ± 0.6	1.7 ± 0.2	− 1.0 ± 0.5	1.0 ± 0.2	− 0.4 ± 0.6	− 0.2 ± 0.6	− 4.8 ± 0.2
MAD	1.8 ± 0.2	2.8 ± 0.3	2.3 ± 0.2	2.2 ± 0.3	2.6 ± 0.2	2.5 ± 0.3	2.1 ± 0.3	
RMSD	2.3 ± 0.2	3.4 ± 0.3	3.4 ± 0.2	2.7 ± 0.3	3.5 ± 0.2	3.3 ± 0.3	2.7 ± 0.3	
MSD	0.4 ± 0.2	1.1 ± 0.3	− 0.1 ± 0.3	0.7 ± 0.3	0.0 ± 0.3	0.0 ± 0.3	0.4 ± 0.3	
Max	4.3 ± 0.3	6.6 ± 0.8	6.4 ± 0.3	4.8 ± 0.6	6.7 ± 0.4	6.4 ± 0.7	4.7 ± 0.7	
*R* ^2^	0.79 ± 0.03	0.54 ± 0.07	0.61 ± 0.04	0.71 ± 0.06	0.55 ± 0.04	0.60 ± 0.06	0.71 ± 0.06	
τ_r_	1.00 ± 0.16	0.67 ± 0.10	0.33 ± 0.08	1.00 ± 0.13	0.33 ± 0.10	1.00 ± 0.18	1.00 ± 0.22	
τ_r90_	1.00 ± 0.04	0.60 ± 0.02	0.33 ± 0.08	1.00 ± 0.13	0.60 ± 0.04	1.00 ± 0.00	1.00 ± 0.08	

Experimental relative affinities are given in the last column [38]

Six different sets of FEP calculations were performed to see how the results changed with variations in the computational method. First, three different sets of charges were employed for the ligands: They were obtained either with the RESP method, based on Hartree–Fock/6–31G* calculations, or with the cheaper AM1-BCC approach. In the former case, geometries were first optimised at the Hartree–Fock/6–31G level of theory (RH charge set). In the latter case, we either used the same geometries (BH) or geometries optimised with the semiempirical AM1 method (BA). Moreover, in the FEP calculations, we included in the perturbed group either only atoms directly involved in the perturbation (SP), i.e. those in the para substituent or all atoms of the terminal substituted tetrafluorophenyl group (LP).

The results (∆∆*G*_bind_) of all calculations are shown in Table 1. This table also contains seven quality estimates, viz. the mean absolute deviation (MAD), the root-mean-square deviation (RMSD), the mean singed deviation (MSD), the maximum error (Max), the correlation coefficient (*R*^2^), Kendall’s rank correlation coefficient including only the relative energies considered (τ_r_), as well as the same correlation coefficient calculated only for those experimental and calculated energies that are statistically significant at the 90% level (τ_r90_).

The differences between the ∆∆*G*_bind_ results obtained with the small and large perturbed groups (SP and LP) are up to 5 kJ/mol for RH and 2–3 kJ/mol for BA and BH, with MADs of 1–2 kJ/mol. The largest difference is for the OEt → OMe perturbation for both the RH and BA charges. However, owing to the good precision of the simulations (0.2–0.8 kJ/mol), the differences are statistically significant for four (RH) or three of the calculations. Consequently, two (BH) to five (RH) of the quality estimates are also significantly different between the calculations with different perturbed groups. For the RH and BH charges, the SP calculations give the better results, whereas for the BA charges, the opposite is true. Therefore, it is hard to draw any firm conclusions from this variation. Apparently, there are at least two opposing effects for variations in the perturbed group. A larger perturbed group allows for a larger movement of atoms in the ligand, which may lead to improved results if the two groups actually have a different geometry. However, this larger variation in the coordinates also introduces more freedom in the system that may give rise to more random noise. The latter is reflected by a 0.2–0.3 kJ/mol higher uncertainty in all LP results, compared to the SP results. The difference is somewhat larger for RH than for the other two charge sets.

The BA and BH charge sets give very similar results, with differences in the individual ∆∆*G*_bind_ values of less than 1.6 kJ/mol (0.9–1.1 kJ/mol MAD differences). In fact, only one of the 14 values has a statistically significant difference at the 95% level. Likewise, only one of the quality estimates gives a significant difference. On the other hand, the RH charges are more different, giving rise to differences in the ∆∆*G*_bind_ results of up to 6 kJ/mol with MADs of 2 kJ/mol. Two to five of the individual results are significantly different for the four series. This leads to significant differences in nearly all of the quality estimates with the small perturbed group (SP), the RH charges always giving the better results. However, with the large perturbed group (LP), the larger uncertainty in the individual results make the differences in the quality measures statistically significant only in three cases, involving τ_r_ and τ_r90_. In this case, RH/LP always give the worse results.

Thus, again, it seems hard to draw any firm conclusions about the performance of the three charge sets—in fact RH/SP gives the best results among all calculations, whereas RH/LP gives the worse results, perhaps together with BH/SP. It seems that the RESP charges give strongly varying results, simply because they are larger in magnitude than the BCC charges. This prohibits any firm recommendation of any of the charge or perturbed-group methods. Instead, the observed variations in the results and performance of the six approaches have to be seen as a measurement of the sensitivity of the results to various quite reasonable variations in the theoretical method. Quite satisfying, the variation in the quality estimates is not extensive, e.g. 2–3 kJ/mol in the MAD and 0.5–0.8 in *R*^2^. Thus, a study like this can provide an estimate of the stability of the results, in addition to the precision, overlap measures and sampling estimates and therefore provide another approach to generate independent simulations [20] to better estimate the true uncertainty of the calculations.

**Fig. 2:**
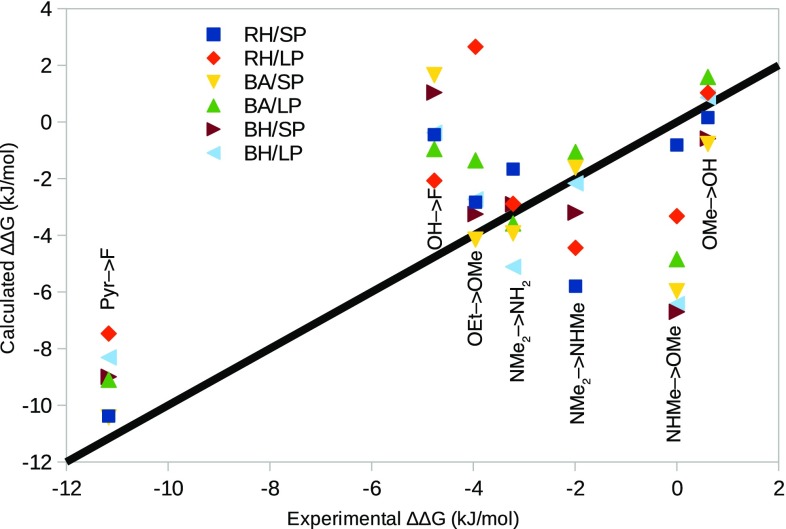
Results of the FEP calculations, compared to the experimental data. The black line represents the perfect correlation

The calculated ∆∆*G*_bind_ values are compared to the experimental results in Fig. 2. It can be seen that for five of the transformations, the range of the six approaches overlap with the ideal correlation, and for the remaining two, at least one of the results come within 1 kJ/mol of the experimental results. A natural choice is to test the consensus (i.e. average) results of the six methods with long simulations. These results are also included in Table 1 (with standard errors calculated from the variation of the results over the six methods). It can be seen that it comes close to the best results for all quality measures, e.g. MAD = 2.1 ± 0.3, *R*^2^ = 0.71 ± 0.06 and τ_r_ = τ_r90_ = 1.0 ± 0.2.

Taking all results together, it can be seen that MAD = 1.8–3.1 kJ/mol. This is a quite typical result of FEP for a well-behaving protein; previous large-scale studies have given MADs of 3–7 kJ/mol [9–12]. The RMSDs are slightly larger, 2.3–3.5 kJ/mol and the maximum error is 4–7 kJ/mol. Although these errors are small in energy terms, the MAD and maximum errors correspond to a factor of 2.1–3.5 and 6–15 in the binding constant, which are quite large from an experimental point of view. *R*^2^ is 0.54–0.79, i.e. of an intermediate quality. However, *R*^2^ is most affected by the Pyr → F transformation, which gives the largest experimental difference and it is underestimated in all calculations; without this perturbation, *R*^2^ is lower, 0.12–0.38. The reason for this is that all the other six relative affinities are less than 5 kJ/mol (in absolute terms), making it a formidable task to obtain an accurate correlation. On the other hand, Kendall’s τ_r_ is perfect for half of the methods (RH/SP, BA/LP and BH/LP), showing that these calculations give the correct sign for all ∆∆*G*_bind_ estimates. The other three methods give one or two incorrect signs, but for BH/SP one of those is not statistically significant. This is arguably the most important quality measure, because during lead optimisation, the prime question is whether a new drug candidate will be better than the previous ones. Therefore, it seems that FEP may be a valuable approach to predict at least the sign of putative drug candidates of galectin-3.

## Conclusions

We have studied the binding of the eight substituted tetrafluorophenyl-triazole-thiogalactoside inhibitors in Fig. 1a to galectin-3 with FEP calculations. The results show that we can reproduce experimental relative binding affinities with a MAD of 2–3 kJ/mol, which is similar or better than FEP results obtained for other proteins [9–12]. This is quite satisfying, especially as we have had problems to reproduce experimental affinities with FEP before for this protein (7 kJ/mol error for a single perturbation obtained with similar methods) [45]. The correlation coefficient is also acceptable, 0.5–0.8, especially considering that six of the seven relative energies are below 5 kJ/mol. Moreover, most approaches give a perfect Kendall’s τ.

We have also studied how the calculated binding affinities change with small variations in the computational method, employing either RESP or AM1-BCC charges for the ligands, optimising it with two different QM methods before the charge calculation and employing different sizes of the perturbed group in the FEP calculations (only the differing atoms or the whole substituted tetrafluorophenyl group). We show that the variations give only rather small differences in the calculated relative affinities and that none of the approaches is consistently and significantly better than the others. Instead, we suggest that such small, but reasonable variations in the theoretical method could be employed to assess the stability of the results obtained.

Recently, it has been argued that the results of a single FEP calculation of binding energies is quite uncertain and that the precision estimated by standard methods underestimates the true uncertainty [22]. Instead, the true uncertainty should be estimated by performing a number of independent simulations, using different starting conditions. This has also repeatedly been suggested for other types of calculations based on MD simulations [15–21].

Alternatively, thermodynamic cycle-closure hysteresis has been suggested as a cheaper estimate of the uncertainty of the calculations [67, 68]. However, it is a rather blunt estimate, because it can give a good result by chance or by cancellation of errors. To be useful, all ligands should be involved in at least one cycle, requiring almost the same number of extra calculations as a set of independent simulations. Moreover, they normally require larger perturbations, because the original scheme (without cycles) is typically designed to make the perturbations as small as possible. Moreover, the cycle-closure hysteresis is independent of errors in the force field and therefore ignores method errors considered by the present approach.

Traditionally, independent simulations have been generated by using different random seeds for the starting velocities. We have employed other arbitrary choices in the setup, in particular the position of solvation water molecules, but also the selection of alternative conformations or the protonation of the residues in the protein [20, 23–25]. It is a natural extension to include also variations in the computational method. In analogy with the naming of the other approaches to obtain independent simulations [20], we suggest that this should be called methods-induced independent trajectories (MIIT). However, as for the selection of conformations and protonations, it needs to be checked that the different approaches do not give rise to results that are significantly different. Still, it is important in computational studies to ensure that the results are not sensitive to reasonable variations in the computational setup.

## Electronic supplementary material

Below is the link to the electronic supplementary material.

Supplementary material 1 (DOCX 107 KB)
