# Supplementary material for: Assessing the stability of free-energy perturbation calculations by performing variations in the method
Source: J Comput Aided Mol Des. 2018 Mar 13;32(4):529–36. doi: 10.1007/s10822-018-0110-5 (PMC5889414; doi:10.1007/s10822-018-0110-5)
Supplement: Supplementary file 1 — Supplementary material 1 (DOCX 107 KB) [file 10822_2018_110_MOESM1_ESM.docx]

*Supplementary Material*

Assessing the stability of free-energy perturbation calculations by performing variations in the method

Francesco Manzoni and Ulf Ryde

Theoretical Chemistry, Department of Chemistry, Lund University, Chemical Centre,
P. O. Box 124, 221 00 Lund, Sweden

Correspondence to Ulf Ryde, E-mail: Ulf.Ryde@teokem.lu.se,
Tel: +46 – 46 2224502, Fax: +46 – 46 2228648

2018-02-21

Results of the short simulations

Initially, two of the perturbations were run with shorter simulation times, viz. 0.5 ns equilibration and 1 ns production simulations. The results of these simulations are shown in Table S1 and they are compared to the corresponding results with long (1 + 5 ns) simulations, as well as with experimental results [67]. It can be seen that the most prominent difference is the appreciably larger uncertainty in the short simulations: 1.2–1.6 kJ/mol, compared to 0.5–0.7 kJ/mol for the corresponding long simulations. The latter estimates are more similar to, but still somewhat larger than the uncertainty in the experimental data, 0.2–0.4 kJ/mol. Moreover, it can be seen that there are rather large differences in the results (∆∆*G*_bind_) of the individual simulations, up to 4 kJ/mol and 2 kJ/mol on average. However, only one (BA/LP) or three (BH/LP) of the seven differences are significant with a 95% confidence (owing to the rather poor precision of the short simulations). Most important, there are no statistically significant differences in any of the seven considered quality estimates of the two sets of simulations. However, the overlap measures often indicated that the overlap is somewhat poor (e.g. Ω < 0.85, Π < 0.5, *w*_max_ > 0.5) for many of the short simulations. Therefore, we will in the main article discuss only the results of the long simulations.

Table S1. Comparison of the results obtained with short and long simulations. Experimental relative affinities are given in the last column [67].

|  | BA/LP | | BH/LP | | Exp. |
| --- | --- | --- | --- | --- | --- |
|  | Long | Short | Long | Short |  |
| OMe→OH | 1.6±0.6 | 1.3±1.4 | 0.9±0.6 | 0.7±1.4 | 0.6±0.3 |
| NHMe→OMe | -4.8±0.6 | -5.4±1.3 | -6.4±0.6 | -5.5±1.4 | 0.0±0.3 |
| NMe_2_→NHMe | -1.1±0.6 | -0.1±1.4 | -2.2±0.7 | 1.1±1.5 | -2.0±0.2 |
| NMe_2_→NH_2_ | -3.6±0.7 | -5.9±1.5 | -5.1±0.7 | -0.8±1.6 | -3.2±0.2 |
| OEt→OMe | -1.4±0.6 | -3.1±1.4 | -2.7±0.6 | -2.6±1.4 | -4.0±0.4 |
| Pyr→F | -9.1±0.7 | -8.6±1.6 | -8.3±0.7 | -7.8±1.6 | -11.2 |
| OH→F | -1.0±0.5 | -5.4±1.2 | -0.4±0.6 | -4.8±1.2 | -4.8±0.2 |
| MAD | 2.2±0.3 | 2.4±0.5 | 2.5±0.3 | 2.4±0.5 |  |
| RMSD | 2.7±0.3 | 2.6±0.5 | 3.3±0.3 | 2.7±0.5 |  |
| MSD | 0.7±0.3 | -0.3±0.6 | 0.0±0.3 | 0.7±0.6 |  |
| Max | 4.8±0.6 | 3.6±1.0 | 6.4±0.7 | 3.9±1.1 |  |
| *R*^2^ | 0.71±0.06 | 0.77±0.09 | 0.60±0.06 | 0.75±0.11 |  |
| τ_r_ | 1.00±0.13 | 1.00±0.18 | 1.00±0.18 | 0.71±0.23 |  |
| τ_r90_ | 1.00±0.13 | 1.00±0.05 | 1.00±0.00 | 1.00±0.10 |  |

Table S2. Amber topology files for the eight ligands for the three charge sets. The last column shows the charges.

RH charges

0 0 2

F ligand with RH charges

molecule.res

L1b INT 0

CORRECT OMIT DU BEG

0.0000

1 DUMM DU M 0 -1 -2 0.000 .0 .0 .00000

2 DUMM DU M 1 0 -1 1.449 .0 .0 .00000

3 DUMM DU M 2 1 0 1.523 111.21 .0 .00000

4 F4 f M 3 2 1 1.540 111.208 -180.000 -0.143199

5 C14 ca M 4 3 2 1.374 141.140 46.175 0.062331

6 C15 ca S 5 4 3 1.393 119.955 157.514 0.352938

7 F5 f E 6 5 4 1.374 116.776 0.761 -0.186687

8 C13 ca M 5 4 3 1.391 119.589 -22.946 0.213022

9 F3 f E 8 5 4 1.372 120.463 0.337 -0.150294

10 C12 ca M 8 5 4 1.390 119.165 -179.470 0.062331

11 F2 f E 10 8 5 1.375 120.110 179.951 -0.143199

12 C11 ca M 10 8 5 1.389 119.614 -0.207 0.352938

13 F1 f E 12 10 8 1.385 117.276 -179.859 -0.186687

14 C10 ca M 12 10 8 1.405 123.111 -0.055 -0.557561

15 C9 cd M 14 12 10 1.460 121.105 -179.631 0.715335

16 N3 nd S 15 14 12 1.388 122.286 163.734 -0.350777

17 N2 nc E 16 15 14 1.315 109.112 -179.763 -0.157148

18 C8 cc M 15 14 12 1.394 129.509 -15.849 -0.543215

19 H11 h4 E 18 15 14 1.073 132.385 -2.710 0.310673

20 N1 na M 18 15 14 1.358 105.329 179.703 0.271960

21 C7 c3 M 20 18 15 1.473 128.497 179.554 -0.116401

22 C6 c3 B 21 20 18 1.538 111.380 -70.848 0.346549

23 O4 oh S 22 21 20 1.448 106.047 60.387 -0.650556

24 H8 ho E 23 22 21 0.980 109.793 178.512 0.373053

25 H9 h1 E 22 21 20 1.097 109.416 -60.631 0.146695

26 H10 h1 E 21 20 18 1.098 106.486 45.924 0.164950

27 C3 c3 M 21 20 18 1.549 111.540 164.196 -0.170575

28 O2 oh S 27 21 20 1.442 111.772 60.650 -0.572260

29 H4 ho E 28 27 21 0.987 107.703 -63.795 0.395149

30 H5 h1 E 27 21 20 1.100 108.341 -61.825 0.133373

31 C2 c3 M 27 21 20 1.532 109.342 179.912 0.282105

32 C1 c3 3 31 27 21 1.524 114.062 -172.043 0.166972

33 O1 oh S 32 31 27 1.450 110.298 178.705 -0.677243

34 H1 ho E 33 32 31 0.980 107.813 -65.744 0.418478

35 H2 h1 E 32 31 27 1.092 110.210 -64.418 0.071181

36 H3 h1 E 32 31 27 1.093 108.960 55.272 0.071181

37 H6 h1 E 31 27 21 1.101 109.543 64.630 0.041112

38 O3 os M 31 27 21 1.476 110.569 -54.610 -0.333901

39 C4 c3 M 38 31 27 1.442 113.463 58.855 -0.116534

40 H7 h2 E 39 38 31 1.099 111.174 62.626 0.142943

41 S1 ss M 39 38 31 1.886 108.188 -179.992 -0.198394

42 C5 ca M 41 39 38 1.855 100.404 -89.660 0.089175

43 C16 ca M 42 41 39 1.399 118.660 -135.793 -0.076889

44 H12 ha E 43 42 41 1.084 120.213 2.784 0.141281

45 C18 ca M 43 42 41 1.398 119.641 -177.693 -0.340310

46 H14 ha E 45 43 42 1.086 119.294 -179.880 0.184247

47 C20 ca M 45 43 42 1.406 121.222 -0.766 0.285200

48 C21 c3 3 47 45 43 1.512 121.003 179.138 -0.296918

49 H16 hc E 48 47 45 1.095 111.517 148.284 0.088415

50 H17 hc E 48 47 45 1.099 111.174 -92.152 0.088415

51 H18 hc E 48 47 45 1.096 111.467 27.480 0.088415

52 C19 ca M 47 45 43 1.406 118.079 0.245 -0.340310

53 H15 ha E 52 47 45 1.086 119.525 -179.832 0.184247

54 C17 ca M 52 47 45 1.399 121.354 0.861 -0.076889

55 H13 ha E 54 52 47 1.084 120.220 178.810 0.141281

LOOP

C10 C15

N1 N2

C4 C6

C17 C5

IMPROPER

C15 C13 C14 F4

C10 C14 C15 F5

C14 C12 C13 F3

C11 C13 C12 F2

C10 C12 C11 F1

C11 C15 C10 C9

C10 C8 C9 N3

C9 H11 C8 N1

C7 C8 N1 N2

C16 C17 C5 S1

C5 C18 C16 H12

C16 C20 C18 H14

C21 C18 C20 C19

C20 C17 C19 H15

C5 C19 C17 H13

DONE

STOP

0 0 2

OMe ligand with RH charges

molecule.res

L1C INT 0

CORRECT OMIT DU BEG

0.0000

1 DUMM DU M 0 -1 -2 0.000 .0 .0 .00000

2 DUMM DU M 1 0 -1 1.449 .0 .0 .00000

3 DUMM DU M 2 1 0 1.523 111.21 .0 .00000

4 F3 f M 3 2 1 1.540 111.208 -180.000 -0.166450

5 C14 ca M 4 3 2 1.390 138.736 43.144 0.031464

6 C15 ca S 5 4 3 1.394 117.623 161.034 0.389259

7 F4 f E 6 5 4 1.375 116.537 1.048 -0.194467

8 C13 ca M 5 4 3 1.399 120.323 -19.266 0.229971

9 O5 os S 8 5 4 1.368 127.903 0.696 -0.303656

10 C16 c3 3 9 8 5 1.470 121.983 -11.254 -0.022092

11 H12 h1 E 10 9 8 1.088 103.711 -173.727 0.095316

12 H13 h1 E 10 9 8 1.093 110.743 -55.155 0.095316

13 H14 h1 E 10 9 8 1.091 110.911 67.461 0.095316

14 C12 ca M 8 5 4 1.405 116.092 179.704 0.031464

15 F2 f E 14 8 5 1.377 119.248 179.972 -0.166450

16 C11 ca M 14 8 5 1.384 121.297 0.105 0.389259

17 F1 f E 16 14 8 1.388 117.260 179.934 -0.194467

18 C10 ca M 16 14 8 1.406 123.420 0.088 -0.633508

19 C9 cd M 18 16 14 1.459 121.527 179.990 0.753589

20 N3 nd S 19 18 16 1.388 122.450 164.907 -0.377611

21 N2 nc E 20 19 18 1.317 109.098 -179.805 -0.135969

22 C8 cc M 19 18 16 1.394 129.401 -14.600 -0.537212

23 H11 h4 E 22 19 18 1.073 132.256 -2.730 0.309279

24 N1 na M 22 19 18 1.359 105.423 179.687 0.235502

25 C7 c3 M 24 22 19 1.472 128.517 179.347 -0.099859

26 C6 c3 B 25 24 22 1.538 111.423 -72.440 0.337911

27 O4 oh S 26 25 24 1.449 105.962 60.643 -0.650878

28 H8 ho E 27 26 25 0.980 109.781 179.308 0.375626

29 H9 h1 E 26 25 24 1.095 109.443 -60.416 0.149390

30 H10 h1 E 25 24 22 1.097 106.488 44.399 0.164463

31 C3 c3 M 25 24 22 1.549 111.482 162.639 -0.165876

32 O2 oh S 31 25 24 1.441 111.891 60.607 -0.568473

33 H4 ho E 32 31 25 0.988 107.536 -62.388 0.386613

34 H5 h1 E 31 25 24 1.101 108.293 -61.874 0.132866

35 C2 c3 M 31 25 24 1.532 109.340 179.944 0.266982

36 C1 c3 3 35 31 25 1.525 114.062 -172.012 0.176243

37 O1 oh S 36 35 31 1.451 110.329 178.298 -0.678574

38 H1 ho E 37 36 35 0.979 107.643 -65.217 0.417769

39 H2 h1 E 36 35 31 1.092 110.204 -64.785 0.068795

40 H3 h1 E 36 35 31 1.093 108.948 54.934 0.068795

41 H6 h1 E 35 31 25 1.101 109.591 64.586 0.042702

42 O3 os M 35 31 25 1.476 110.550 -54.687 -0.327423

43 C4 c3 M 42 35 31 1.443 113.429 58.897 -0.129873

44 H7 h2 E 43 42 35 1.098 111.178 62.630 0.149875

45 S1 ss M 43 42 35 1.887 108.142 179.908 -0.197275

46 C5 ca M 45 43 42 1.853 100.389 -89.484 0.087231

47 C17 ca M 46 45 43 1.399 118.652 -135.953 -0.077081

48 H15 ha E 47 46 45 1.084 120.176 2.751 0.141461

49 C19 ca M 47 46 45 1.399 119.650 -177.802 -0.339173

50 H17 ha E 49 47 46 1.086 119.289 -179.945 0.183593

51 C21 ca M 49 47 46 1.405 121.213 -0.742 0.283313

52 C22 c3 3 51 49 47 1.513 120.999 179.119 -0.293504

53 H19 hc E 52 51 49 1.095 111.459 148.113 0.087235

54 H20 hc E 52 51 49 1.098 111.174 -92.337 0.087235

55 H21 hc E 52 51 49 1.095 111.435 27.356 0.087235

56 C20 ca M 51 49 47 1.406 118.103 0.179 -0.339173

57 H18 ha E 56 51 49 1.086 119.530 -179.854 0.183593

58 C18 ca M 56 51 49 1.398 121.340 0.895 -0.077081

59 H16 ha E 58 56 51 1.085 120.283 178.783 0.141461

LOOP

C10 C15

N1 N2

C4 C6

C18 C5

IMPROPER

C15 C13 C14 F3

C10 C14 C15 F4

C12 C14 C13 O5

C11 C13 C12 F2

C12 C10 C11 F1

C11 C15 C10 C9

C10 C8 C9 N3

C9 H11 C8 N1

C7 C8 N1 N2

C17 C18 C5 S1

C5 C19 C17 H15

C17 C21 C19 H17

C22 C19 C21 C20

C21 C18 C20 H18

C5 C20 C18 H16

DONE

STOP

0 0 2

MHMe ligand with RH charges

molecule.res

L1d INT 0

CORRECT OMIT DU BEG

0.0000

1 DUMM DU M 0 -1 -2 0.000 .0 .0 .00000

2 DUMM DU M 1 0 -1 1.449 .0 .0 .00000

3 DUMM DU M 2 1 0 1.523 111.21 .0 .00000

4 F3 f M 3 2 1 1.540 111.208 -180.000 -0.179667

5 C14 ca M 4 3 2 1.391 138.183 44.825 0.106435

6 C15 ca S 5 4 3 1.392 117.892 160.447 0.341494

7 F4 f E 6 5 4 1.376 116.448 0.698 -0.194329

8 C13 ca M 5 4 3 1.406 119.650 -20.044 -0.020744

9 N4 nh B 8 5 4 1.370 126.327 0.414 -0.244598

10 H19 hn E 9 8 5 1.007 115.442 179.803 0.264572

11 C22 c3 3 9 8 5 1.464 126.664 -0.084 -0.202660

12 H20 h1 E 11 9 8 1.092 107.611 -179.913 0.112348

13 H21 h1 E 11 9 8 1.094 111.865 -60.719 0.112348

14 H22 h1 E 11 9 8 1.094 111.859 60.996 0.112348

15 C12 ca M 8 5 4 1.410 114.505 -179.441 0.106435

16 F2 f E 15 8 5 1.394 117.168 179.912 -0.179667

17 C11 ca M 15 8 5 1.380 122.782 -0.286 0.341494

18 F1 f E 17 15 8 1.390 117.478 -179.812 -0.194329

19 C10 ca M 17 15 8 1.406 123.010 0.080 -0.558365

20 C9 cd M 19 17 15 1.459 121.669 -179.697 0.702907

21 N3 nd S 20 19 17 1.388 122.540 164.242 -0.351656

22 N2 nc E 21 20 19 1.318 109.112 -179.803 -0.164548

23 C8 cc M 20 19 17 1.395 129.372 -15.247 -0.550942

24 H11 h4 E 23 20 19 1.072 132.182 -2.748 0.305541

25 N1 na M 23 20 19 1.359 105.433 179.563 0.274395

26 C7 c3 M 25 23 20 1.472 128.563 179.415 -0.123980

27 C6 c3 B 26 25 23 1.538 111.514 -72.938 0.359244

28 O4 oh S 27 26 25 1.448 105.992 60.750 -0.651660

29 H8 ho E 28 27 26 0.980 109.791 -179.986 0.373424

30 H9 h1 E 27 26 25 1.096 109.331 -60.308 0.143285

31 H10 h1 E 26 25 23 1.098 106.473 43.820 0.164957

32 C3 c3 M 26 25 23 1.549 111.479 162.035 -0.159326

33 O2 oh S 32 26 25 1.441 111.881 60.613 -0.572953

34 H4 ho E 33 32 26 0.989 107.426 -61.722 0.391199

35 H5 h1 E 32 26 25 1.101 108.234 -61.875 0.131468

36 C2 c3 M 32 26 25 1.532 109.417 179.989 0.266632

37 C1 c3 3 36 32 26 1.524 114.061 -171.976 0.173499

38 O1 oh S 37 36 32 1.451 110.328 178.141 -0.678013

39 H1 ho E 38 37 36 0.979 107.663 -65.042 0.417254

40 H2 h1 E 37 36 32 1.093 110.260 -64.948 0.069228

41 H3 h1 E 37 36 32 1.093 108.952 54.805 0.069228

42 H6 h1 E 36 32 26 1.100 109.538 64.556 0.042689

43 O3 os M 36 32 26 1.476 110.536 -54.635 -0.325039

44 C4 c3 M 43 36 32 1.443 113.482 58.786 -0.133540

45 H7 h2 E 44 43 36 1.098 111.100 62.731 0.149068

46 S1 ss M 44 43 36 1.887 108.118 179.968 -0.199069

47 C5 ca M 46 44 43 1.853 100.432 -89.184 0.084153

48 C16 ca M 47 46 44 1.399 118.690 -136.390 -0.073085

49 H12 ha E 48 47 46 1.085 120.131 2.803 0.140084

50 C18 ca M 48 47 46 1.399 119.701 -177.764 -0.341150

51 H14 ha E 50 48 47 1.086 119.273 -179.904 0.183252

52 C20 ca M 50 48 47 1.405 121.221 -0.728 0.284278

53 C21 c3 3 52 50 48 1.513 121.029 179.114 -0.291888

54 H16 hc E 53 52 50 1.095 111.498 148.334 0.086283

55 H17 hc E 53 52 50 1.099 111.168 -92.026 0.086283

56 H18 hc E 53 52 50 1.096 111.423 27.536 0.086283

57 C19 ca M 52 50 48 1.406 118.082 0.204 -0.341150

58 H15 ha E 57 52 50 1.086 119.533 -179.848 0.183252

59 C17 ca M 57 52 50 1.398 121.338 0.883 -0.073085

60 H13 ha E 59 57 52 1.085 120.257 178.806 0.140084

LOOP

C10 C15

N1 N2

C4 C6

C17 C5

IMPROPER

C15 C13 C14 F3

C10 C14 C15 F4

C12 C14 C13 N4

C22 C13 N4 H19

C11 C13 C12 F2

C12 C10 C11 F1

C11 C15 C10 C9

C10 C8 C9 N3

C9 H11 C8 N1

C7 C8 N1 N2

C16 C17 C5 S1

C5 C18 C16 H12

C16 C20 C18 H14

C21 C18 C20 C19

C20 C17 C19 H15

C5 C19 C17 H13

DONE

STOP

0 0 2

NMe2 ligand with RH charges

molecule.res

L1e INT 0

CORRECT OMIT DU BEG

0.0000

1 DUMM DU M 0 -1 -2 0.000 .0 .0 .00000

2 DUMM DU M 1 0 -1 1.449 .0 .0 .00000

3 DUMM DU M 2 1 0 1.523 111.21 .0 .00000

4 F3 f M 3 2 1 1.540 111.208 -180.000 -0.182989

5 C14 ca M 4 3 2 1.388 138.135 36.796 0.098923

6 C15 ca S 5 4 3 1.389 117.665 164.409 0.387714

7 F4 f E 6 5 4 1.377 116.714 2.175 -0.206136

8 C13 ca M 5 4 3 1.413 119.055 -14.501 -0.109672

9 N4 nh B 8 5 4 1.382 123.070 -2.262 0.113522

10 C22 c3 3 9 8 5 1.468 122.277 145.596 -0.251073

11 H22 h1 E 10 9 8 1.097 108.699 -135.002 0.101172

12 H23 h1 E 10 9 8 1.097 112.061 105.049 0.101172

13 H24 h1 E 10 9 8 1.089 110.689 -16.224 0.101172

14 C23 c3 3 9 8 5 1.468 122.383 -34.615 -0.251073

15 H19 h1 E 14 9 8 1.098 108.753 -134.208 0.101172

16 H20 h1 E 14 9 8 1.089 110.697 -15.335 0.101172

17 H21 h1 E 14 9 8 1.097 111.969 105.813 0.101172

18 C12 ca M 8 5 4 1.411 113.913 177.920 0.098923

19 F2 f E 18 8 5 1.390 119.509 177.337 -0.182989

20 C11 ca M 18 8 5 1.385 122.478 -1.140 0.387714

21 F1 f E 20 18 8 1.390 117.131 -179.960 -0.206136

22 C10 ca M 20 18 8 1.404 123.562 2.052 -0.599405

23 C9 cd M 22 20 18 1.459 121.752 179.121 0.710675

24 N3 nd S 23 22 20 1.388 122.516 165.650 -0.355803

25 N2 nc E 24 23 22 1.318 109.122 -179.989 -0.155536

26 C8 cc M 23 22 20 1.394 129.377 -14.061 -0.540287

27 H11 h4 E 26 23 22 1.072 132.209 -2.577 0.308378

28 N1 na M 26 23 22 1.359 105.451 179.801 0.263273

29 C7 c3 M 28 26 23 1.471 128.569 179.397 -0.123182

30 C6 c3 B 29 28 26 1.538 111.459 -72.437 0.364564

31 O4 oh S 30 29 28 1.448 106.049 60.652 -0.655811

32 H8 ho E 31 30 29 0.979 109.881 179.813 0.373913

33 H9 h1 E 30 29 28 1.096 109.399 -60.389 0.140769

34 H10 h1 E 29 28 26 1.097 106.503 44.322 0.164562

35 C3 c3 M 29 28 26 1.550 111.485 162.615 -0.164233

36 O2 oh S 35 29 28 1.441 111.841 60.516 -0.571638

37 H4 ho E 36 35 29 0.988 107.438 -61.989 0.389858

38 H5 h1 E 35 29 28 1.101 108.208 -61.921 0.133362

39 C2 c3 M 35 29 28 1.532 109.371 179.897 0.266822

40 C1 c3 3 39 35 29 1.524 114.046 -171.972 0.171722

41 O1 oh S 40 39 35 1.451 110.378 178.109 -0.677794

42 H1 ho E 41 40 39 0.979 107.656 -65.141 0.417811

43 H2 h1 E 40 39 35 1.093 110.271 -64.921 0.070423

44 H3 h1 E 40 39 35 1.093 108.908 54.752 0.070423

45 H6 h1 E 39 35 29 1.102 109.545 64.632 0.043423

46 O3 os M 39 35 29 1.476 110.588 -54.583 -0.331441

47 C4 c3 M 46 39 35 1.442 113.466 58.817 -0.112985

48 H7 h2 E 47 46 39 1.098 111.203 62.692 0.141835

49 S1 ss M 47 46 39 1.888 108.101 179.863 -0.203487

50 C5 ca M 49 47 46 1.853 100.323 -89.263 0.085847

51 C16 ca M 50 49 47 1.399 118.668 -136.219 -0.073405

52 H12 ha E 51 50 49 1.084 120.143 2.854 0.139841

53 C18 ca M 51 50 49 1.399 119.641 -177.818 -0.340754

54 H14 ha E 53 51 50 1.087 119.250 -179.793 0.183854

55 C20 ca M 53 51 50 1.405 121.213 -0.740 0.281417

56 C21 c3 3 55 53 51 1.513 120.973 179.139 -0.287283

57 H16 hc E 56 55 53 1.095 111.522 148.697 0.085658

58 H17 hc E 56 55 53 1.099 111.191 -91.731 0.085658

59 H18 hc E 56 55 53 1.095 111.487 27.877 0.085658

60 C19 ca M 55 53 51 1.405 118.122 0.229 -0.340754

61 H15 ha E 60 55 53 1.086 119.530 -179.931 0.183854

62 C17 ca M 60 55 53 1.398 121.318 0.883 -0.073405

63 H13 ha E 62 60 55 1.084 120.220 178.774 0.139841

LOOP

C10 C15

N1 N2

C4 C6

C17 C5

IMPROPER

C15 C13 C14 F3

C10 C14 C15 F4

C12 C14 C13 N4

C23 C22 N4 C13

C11 C13 C12 F2

C12 C10 C11 F1

C11 C15 C10 C9

C10 C8 C9 N3

C9 H11 C8 N1

C7 C8 N1 N2

C16 C17 C5 S1

C5 C18 C16 H12

C16 C20 C18 H14

C21 C18 C20 C19

C20 C17 C19 H15

C5 C19 C17 H13

DONE

STOP

0 0 2

OEt ligand with RH charges

molecule.res

L1G INT 0

CORRECT OMIT DU BEG

0.0000

1 DUMM DU M 0 -1 -2 0.000 .0 .0 .00000

2 DUMM DU M 1 0 -1 1.449 .0 .0 .00000

3 DUMM DU M 2 1 0 1.523 111.21 .0 .00000

4 C17 c3 M 3 2 1 1.540 111.208 -180.000 -0.365059

5 H18 hc E 4 3 2 1.096 25.123 -157.901 0.102724

6 H19 hc E 4 3 2 1.093 94.721 76.576 0.102724

7 H20 hc E 4 3 2 1.094 94.509 -32.378 0.102724

8 C16 c3 M 4 3 2 1.517 134.875 -157.515 0.323033

9 H12 h1 E 8 4 3 1.092 112.348 60.268 0.026020

10 H13 h1 E 8 4 3 1.095 112.271 -63.019 0.026020

11 O5 os M 8 4 3 1.486 105.585 178.702 -0.317598

12 C13 ca M 11 8 4 1.368 122.019 168.417 0.159957

13 C14 ca B 12 11 8 1.400 127.375 20.307 0.060201

14 F3 f E 13 12 11 1.389 120.164 -0.311 -0.173520

15 C15 ca S 13 12 11 1.394 122.043 178.838 0.376700

16 F4 f E 15 13 12 1.377 116.588 -179.042 -0.194624

17 C12 ca M 12 11 8 1.405 116.501 -161.776 0.060201

18 F2 f E 17 12 11 1.377 119.263 1.790 -0.173520

19 C11 ca M 17 12 11 1.384 121.309 -178.725 0.376700

20 F1 f E 19 17 12 1.388 117.329 -179.675 -0.194624

21 C10 ca M 19 17 12 1.406 123.431 -0.240 -0.615110

22 C9 cd M 21 19 17 1.459 121.562 -179.490 0.744861

23 N3 nd S 22 21 19 1.388 122.410 164.176 -0.383570

24 N2 nc E 23 22 21 1.318 109.060 -179.783 -0.127789

25 C8 cc M 22 21 19 1.394 129.405 -15.315 -0.531038

26 H11 h4 E 25 22 21 1.072 132.252 -2.745 0.309535

27 N1 na M 25 22 21 1.359 105.382 179.652 0.229778

28 C7 c3 M 27 25 22 1.472 128.492 179.292 -0.101349

29 C6 c3 B 28 27 25 1.538 111.353 -72.479 0.358655

30 O4 oh S 29 28 27 1.448 105.983 60.561 -0.656289

31 H8 ho E 30 29 28 0.979 109.865 179.457 0.375296

32 H9 h1 E 29 28 27 1.095 109.505 -60.557 0.143090

33 H10 h1 E 28 27 25 1.097 106.494 44.293 0.163076

34 C3 c3 M 28 27 25 1.549 111.558 162.637 -0.173814

35 O2 oh S 34 28 27 1.442 111.864 60.564 -0.567056

36 H4 ho E 35 34 28 0.988 107.483 -62.215 0.385150

37 H5 h1 E 34 28 27 1.101 108.263 -61.887 0.135096

38 C2 c3 M 34 28 27 1.532 109.353 179.898 0.268027

39 C1 c3 3 38 34 28 1.524 114.025 -172.031 0.168606

40 O1 oh S 39 38 34 1.451 110.328 178.279 -0.677215

41 H1 ho E 40 39 38 0.980 107.690 -65.278 0.417666

42 H2 h1 E 39 38 34 1.093 110.308 -64.811 0.071201

43 H3 h1 E 39 38 34 1.093 108.925 54.908 0.071201

44 H6 h1 E 38 34 28 1.101 109.491 64.640 0.043712

45 O3 os M 38 34 28 1.475 110.604 -54.649 -0.330285

46 C4 c3 M 45 38 34 1.443 113.470 58.820 -0.119796

47 H7 h2 E 46 45 38 1.098 111.168 62.707 0.143845

48 S1 ss M 46 45 38 1.886 108.150 179.924 -0.201576

49 C5 ca M 48 46 45 1.854 100.377 -89.377 0.088780

50 C18 ca M 49 48 46 1.398 118.679 -136.013 -0.076224

51 H14 ha E 50 49 48 1.085 120.164 2.665 0.141174

52 C20 ca M 50 49 48 1.399 119.693 -177.844 -0.340327

53 H16 ha E 52 50 49 1.086 119.289 -179.940 0.184197

54 C22 ca M 52 50 49 1.405 121.166 -0.775 0.282582

55 C23 c3 3 54 52 50 1.513 120.970 179.153 -0.291908

56 H21 hc E 55 54 52 1.095 111.478 148.489 0.086980

57 H22 hc E 55 54 52 1.098 111.173 -92.001 0.086980

58 H23 hc E 55 54 52 1.095 111.485 27.609 0.086980

59 C21 ca M 54 52 50 1.405 118.113 0.201 -0.340327

60 H17 ha E 59 54 52 1.087 119.477 -179.865 0.184197

61 C19 ca M 59 54 52 1.399 121.369 0.881 -0.076224

62 H15 ha E 61 59 54 1.084 120.245 178.757 0.141174

LOOP

C10 C15

N1 N2

C4 C6

C19 C5

IMPROPER

C14 C12 C13 O5

C13 C15 C14 F3

C14 C10 C15 F4

C13 C11 C12 F2

C12 C10 C11 F1

C15 C11 C10 C9

C10 C8 C9 N3

C9 H11 C8 N1

C7 C8 N1 N2

C18 C19 C5 S1

C5 C20 C18 H14

C18 C22 C20 H16

C23 C20 C22 C21

C22 C19 C21 H17

C5 C21 C19 H15

DONE

STOP

0 0 2

Pyr ligand with RH charges

molecule.res

L1l INT 0

CORRECT OMIT DU BEG

0.0000

1 DUMM DU M 0 -1 -2 0.000 .0 .0 .00000

2 DUMM DU M 1 0 -1 1.449 .0 .0 .00000

3 DUMM DU M 2 1 0 1.523 111.21 .0 .00000

4 C21 c3 M 3 2 1 1.540 111.208 -180.000 -0.299132

5 H16 hc E 4 3 2 1.095 97.979 108.218 0.088668

6 H17 hc E 4 3 2 1.099 148.231 -108.967 0.088668

7 H18 hc E 4 3 2 1.096 82.027 1.056 0.088668

8 C20 ca M 4 3 2 1.513 39.518 -137.526 0.282236

9 C19 ca B 8 4 3 1.405 120.892 -108.897 -0.339265

10 C17 ca S 9 8 4 1.399 121.367 -178.067 -0.074944

11 H13 ha E 10 9 8 1.084 120.269 178.817 0.139769

12 H15 ha E 9 8 4 1.087 119.501 1.283 0.183352

13 C18 ca M 8 4 3 1.405 120.998 72.254 -0.339265

14 H14 ha E 13 8 4 1.086 119.503 -1.700 0.183352

15 C16 ca M 13 8 4 1.398 121.179 179.087 -0.074944

16 H12 ha E 15 13 8 1.086 120.184 178.636 0.139769

17 C5 ca M 15 13 8 1.399 119.726 -0.697 0.086910

18 S1 ss M 17 15 13 1.853 118.710 -177.871 -0.201716

19 C4 c3 M 18 17 15 1.888 100.289 -136.344 -0.112400

20 H7 h2 E 19 18 17 1.098 107.006 30.620 0.144653

21 C6 c3 B 19 18 17 1.535 109.627 149.769 0.334485

22 O4 oh S 21 19 18 1.449 110.440 -67.707 -0.651396

23 H8 ho E 22 21 19 0.979 109.786 62.366 0.376079

24 H9 h1 E 21 19 18 1.095 109.932 56.542 0.149284

25 O3 os M 19 18 17 1.442 108.094 -89.157 -0.330375

26 C2 c3 M 25 19 18 1.476 113.495 179.828 0.268066

27 C1 c3 3 26 25 19 1.523 104.453 -178.230 0.168918

28 O1 oh S 27 26 25 1.451 110.384 57.182 -0.677126

29 H1 ho E 28 27 26 0.979 107.603 -65.072 0.416993

30 H2 h1 E 27 26 25 1.093 110.268 174.118 0.070926

31 H3 h1 E 27 26 25 1.094 108.899 -66.208 0.070926

32 H6 h1 E 26 25 19 1.101 108.234 -61.156 0.042449

33 C3 c3 M 26 25 19 1.532 110.568 58.757 -0.167574

34 O2 oh S 33 26 25 1.442 107.829 67.267 -0.569565

35 H4 ho E 34 33 26 0.989 107.393 178.062 0.388665

36 H5 h1 E 33 26 25 1.100 108.619 -172.502 0.132982

37 C7 c3 M 33 26 25 1.550 109.426 -54.590 -0.108081

38 H10 h1 E 37 33 26 1.097 108.517 -63.009 0.163277

39 N1 na M 37 33 26 1.470 111.480 179.854 0.262678

40 N2 nc S 39 37 33 1.394 121.613 -18.629 -0.158113

41 N3 nd E 40 39 37 1.319 107.425 -179.542 -0.355859

42 C8 cc M 39 37 33 1.359 128.473 162.254 -0.548629

43 H11 h4 E 42 39 37 1.073 122.372 1.385 0.310226

44 C9 cd M 42 39 37 1.395 105.463 179.337 0.708639

45 C10 ca M 44 42 39 1.457 129.362 179.912 -0.585988

46 C15 ca B 45 44 42 1.406 124.327 166.739 0.370164

47 C14 ca S 46 45 44 1.388 122.814 179.235 0.123664

48 F3 f E 47 46 45 1.392 116.799 -177.561 -0.196602

49 F4 f E 46 45 44 1.377 120.649 -0.116 -0.202596

50 C11 ca M 45 44 42 1.404 121.979 -12.962 0.370164

51 F1 f E 50 45 44 1.390 119.173 0.628 -0.202596

52 C12 ca M 50 45 44 1.384 123.952 179.284 0.123664

53 F2 f E 52 50 45 1.393 117.159 -177.460 -0.196602

54 C13 ca M 52 50 45 1.417 122.741 1.371 -0.156585

55 N4 nh M 54 52 50 1.373 123.376 179.388 0.048606

56 C22 c3 M 55 54 52 1.491 124.159 -18.344 -0.087583

57 H19 h1 E 56 55 54 1.093 110.488 -46.916 0.088102

58 H23 h1 E 56 55 54 1.095 110.944 72.748 0.088102

59 C24 c3 M 56 55 54 1.538 103.299 -168.020 -0.075779

60 H22 hc E 59 56 55 1.094 111.896 -153.565 0.049192

61 H26 hc E 59 56 55 1.097 110.357 86.126 0.049192

62 C25 c3 M 59 56 55 1.542 103.258 -31.617 -0.075779

63 H21 hc E 62 59 56 1.093 113.038 160.617 0.049192

64 H24 hc E 62 59 56 1.098 110.161 -78.570 0.049192

65 C23 c3 M 62 59 56 1.537 103.366 39.389 -0.087583

66 H20 h1 E 65 62 59 1.095 111.203 87.754 0.088102

67 H25 h1 E 65 62 59 1.094 112.958 -150.794 0.088102

LOOP

C5 C17

C7 C6

C9 N3

C13 C14

C23 N4

IMPROPER

C21 C18 C20 C19

C20 C17 C19 H15

C5 C19 C17 H13

C16 C20 C18 H14

C18 C5 C16 H12

C16 C17 C5 S1

C7 C8 N1 N2

C9 H11 C8 N1

C10 C8 C9 N3

C15 C11 C10 C9

C10 C14 C15 F4

C15 C13 C14 F3

C10 C12 C11 F1

C11 C13 C12 F2

C14 C12 C13 N4

C22 C23 N4 C13

DONE

STOP

0 0 2

OH ligand with RH charges

molecule.res

L1m INT 0

CORRECT OMIT DU BEG

0.0000

1 DUMM DU M 0 -1 -2 0.000 .0 .0 .00000

2 DUMM DU M 1 0 -1 1.449 .0 .0 .00000

3 DUMM DU M 2 1 0 1.523 111.21 .0 .00000

4 F3 f M 3 2 1 1.540 111.208 -180.000 -0.148814

5 C14 ca M 4 3 2 1.390 142.446 47.535 0.043669

6 C15 ca S 5 4 3 1.388 120.645 156.958 0.354636

7 F4 f E 6 5 4 1.375 117.233 0.674 -0.190702

8 C13 ca M 5 4 3 1.394 116.927 -23.548 0.223597

9 O5 oh S 8 5 4 1.372 122.865 0.321 -0.485259

10 H19 ho E 9 8 5 0.979 110.865 -0.071 0.404577

11 C12 ca M 8 5 4 1.396 117.222 -179.492 0.043669

12 F2 f E 11 8 5 1.375 119.937 179.898 -0.148814

13 C11 ca M 11 8 5 1.388 120.099 -0.170 0.354636

14 F1 f E 13 11 8 1.387 117.099 -179.864 -0.190702

15 C10 ca M 13 11 8 1.407 123.717 -0.074 -0.573918

16 C9 cd M 15 13 11 1.459 121.420 -179.663 0.727356

17 N3 nd S 16 15 13 1.388 122.373 164.132 -0.379999

18 N2 nc E 17 16 15 1.317 109.153 -179.740 -0.127432

19 C8 cc M 16 15 13 1.395 129.468 -15.311 -0.524787

20 H11 h4 E 19 16 15 1.072 132.324 -2.808 0.313088

21 N1 na M 19 16 15 1.359 105.339 179.614 0.231059

22 C7 c3 M 21 19 16 1.472 128.475 179.489 -0.101787

23 C6 c3 B 22 21 19 1.538 111.390 -72.067 0.345790

24 O4 oh S 23 22 21 1.448 105.985 60.548 -0.658412

25 H8 ho E 24 23 22 0.980 109.819 179.090 0.376987

26 H9 h1 E 23 22 21 1.096 109.460 -60.470 0.145956

27 H10 h1 E 22 21 19 1.098 106.521 44.714 0.162585

28 C3 c3 M 22 21 19 1.549 111.523 163.027 -0.167541

29 O2 oh S 28 22 21 1.442 111.835 60.641 -0.568906

30 H4 ho E 29 28 22 0.988 107.548 -62.835 0.385635

31 H5 h1 E 28 22 21 1.100 108.302 -61.852 0.132455

32 C2 c3 M 28 22 21 1.533 109.329 179.909 0.276337

33 C1 c3 3 32 28 22 1.524 113.993 -172.050 0.172121

34 O1 oh S 33 32 28 1.451 110.310 178.572 -0.678295

35 H1 ho E 34 33 32 0.980 107.668 -65.272 0.418008

36 H2 h1 E 33 32 28 1.092 110.306 -64.581 0.069533

37 H3 h1 E 33 32 28 1.094 109.004 55.192 0.069533

38 H6 h1 E 32 28 22 1.101 109.470 64.693 0.040807

39 O3 os M 32 28 22 1.476 110.605 -54.636 -0.335382

40 C4 c3 M 39 32 28 1.442 113.458 58.793 -0.109499

41 H7 h2 E 40 39 32 1.098 111.194 62.757 0.142064

42 S1 ss M 40 39 32 1.886 108.146 -179.930 -0.201855

43 C5 ca M 42 40 39 1.853 100.440 -89.637 0.089424

44 C16 ca M 43 42 40 1.399 118.702 -135.929 -0.077603

45 H12 ha E 44 43 42 1.085 120.117 2.837 0.141417

46 C18 ca M 44 43 42 1.398 119.681 -177.769 -0.338745

47 H14 ha E 46 44 43 1.087 119.246 -179.869 0.183563

48 C20 ca M 46 44 43 1.405 121.220 -0.707 0.283078

49 C21 c3 3 48 46 44 1.513 120.980 179.116 -0.294812

50 H16 hc E 49 48 46 1.095 111.469 148.525 0.087684

51 H17 hc E 49 48 46 1.099 111.155 -91.950 0.087684

52 H18 hc E 49 48 46 1.095 111.442 27.655 0.087684

53 C19 ca M 48 46 44 1.405 118.074 0.207 -0.338745

54 H15 ha E 53 48 46 1.087 119.459 -179.859 0.183563

55 C17 ca M 53 48 46 1.398 121.396 0.836 -0.077603

56 H13 ha E 55 53 48 1.084 120.272 178.712 0.141417

LOOP

C10 C15

N1 N2

C4 C6

C17 C5

IMPROPER

C15 C13 C14 F3

C10 C14 C15 F4

C12 C14 C13 O5

C11 C13 C12 F2

C12 C10 C11 F1

C11 C15 C10 C9

C10 C8 C9 N3

C9 H11 C8 N1

C7 C8 N1 N2

C16 C17 C5 S1

C5 C18 C16 H12

C16 C20 C18 H14

C21 C18 C20 C19

C20 C17 C19 H15

C5 C19 C17 H13

DONE

STOP

0 0 2

NH2 ligand with RH charges

molecule.res

L1n INT 0

CORRECT OMIT DU BEG

0.0000

1 DUMM DU M 0 -1 -2 0.000 .0 .0 .00000

2 DUMM DU M 1 0 -1 1.449 .0 .0 .00000

3 DUMM DU M 2 1 0 1.523 111.21 .0 .00000

4 F3 f M 3 2 1 1.540 111.208 -180.000 -0.160106

5 C14 ca M 4 3 2 1.390 141.042 48.339 0.027347

6 C15 ca S 5 4 3 1.388 120.121 157.340 0.360797

7 F4 f E 6 5 4 1.376 116.976 0.751 -0.191776

8 C13 ca M 5 4 3 1.403 117.282 -23.106 0.256067

9 N4 nh B 8 5 4 1.367 122.130 0.290 -0.831804

10 H19 hn E 9 8 5 1.005 120.165 179.798 0.402618

11 H20 hn E 9 8 5 1.004 120.122 0.000 0.402618

12 C12 ca M 8 5 4 1.402 115.580 -179.412 0.027347

13 F2 f E 12 8 5 1.390 117.815 179.831 -0.160106

14 C11 ca M 12 8 5 1.384 121.727 -0.315 0.360797

15 F1 f E 14 12 8 1.389 117.336 -179.805 -0.191776

16 C10 ca M 14 12 8 1.406 123.210 -0.001 -0.594494

17 C9 cd M 16 14 12 1.460 121.527 -179.597 0.731964

18 N3 nd S 17 16 14 1.387 122.464 163.204 -0.363605

19 N2 nc E 18 17 16 1.318 109.135 -179.676 -0.157284

20 C8 cc M 17 16 14 1.394 129.384 -16.085 -0.557271

21 H11 h4 E 20 17 16 1.073 132.264 -2.860 0.308058

22 N1 na M 20 17 16 1.360 105.409 179.454 0.267456

23 C7 c3 M 22 20 17 1.471 128.520 179.506 -0.118490

24 C6 c3 B 23 22 20 1.538 111.475 -73.104 0.353527

25 O4 oh S 24 23 22 1.449 105.986 60.774 -0.651679

26 H8 ho E 25 24 23 0.979 109.778 179.875 0.372823

27 H9 h1 E 24 23 22 1.096 109.453 -60.243 0.142932

28 H10 h1 E 23 22 20 1.097 106.562 43.715 0.163948

29 C3 c3 M 23 22 20 1.549 111.475 162.019 -0.163767

30 O2 oh S 29 23 22 1.441 111.865 60.578 -0.573370

31 H4 ho E 30 29 23 0.989 107.456 -61.844 0.390836

32 H5 h1 E 29 23 22 1.101 108.223 -61.870 0.132247

33 C2 c3 M 29 23 22 1.532 109.402 179.949 0.273521

34 C1 c3 3 33 29 23 1.525 114.011 -172.052 0.168647

35 O1 oh S 34 33 29 1.451 110.282 178.363 -0.677878

36 H1 ho E 35 34 33 0.979 107.670 -65.073 0.417723

37 H2 h1 E 34 33 29 1.092 110.285 -64.729 0.070529

38 H3 h1 E 34 33 29 1.093 108.930 55.000 0.070529

39 H6 h1 E 33 29 23 1.101 109.534 64.626 0.041032

40 O3 os M 33 29 23 1.475 110.610 -54.685 -0.333507

41 C4 c3 M 40 33 29 1.443 113.468 58.734 -0.108761

42 H7 h2 E 41 40 33 1.098 111.158 62.863 0.141140

43 S1 ss M 41 40 33 1.888 108.111 -179.862 -0.203102

44 C5 ca M 43 41 40 1.853 100.448 -89.392 0.086808

45 C16 ca M 44 43 41 1.398 118.692 -136.260 -0.075911

46 H12 ha E 45 44 43 1.086 120.120 2.858 0.140427

47 C18 ca M 45 44 43 1.398 119.762 -177.731 -0.338389

48 H14 ha E 47 45 44 1.085 119.309 -179.886 0.182828

49 C20 ca M 47 45 44 1.405 121.133 -0.779 0.281953

50 C21 c3 3 49 47 45 1.512 120.960 179.090 -0.292905

51 H16 hc E 50 49 47 1.095 111.506 148.765 0.086836

52 H17 hc E 50 49 47 1.099 111.226 -91.636 0.086836

53 H18 hc E 50 49 47 1.096 111.480 28.016 0.086836

54 C19 ca M 49 47 45 1.405 118.143 0.282 -0.338389

55 H15 ha E 54 49 47 1.086 119.533 -179.904 0.182828

56 C17 ca M 54 49 47 1.398 121.345 0.788 -0.075911

57 H13 ha E 56 54 49 1.085 120.272 178.798 0.140427

LOOP

C10 C15

N1 N2

C4 C6

C17 C5

IMPROPER

C15 C13 C14 F3

C10 C14 C15 F4

C12 C14 C13 N4

C13 H20 N4 H19

C11 C13 C12 F2

C12 C10 C11 F1

C11 C15 C10 C9

C10 C8 C9 N3

C9 H11 C8 N1

C7 C8 N1 N2

C16 C17 C5 S1

C5 C18 C16 H12

C16 C20 C18 H14

C21 C18 C20 C19

C20 C17 C19 H15

C5 C19 C17 H13

DONE

BA charges

0 0 2

F ligand with BA charges

molecule.res

L1b INT 0

CORRECT OMIT DU BEG

0.0000

1 DUMM DU M 0 -1 -2 0.000 .0 .0 .00000

2 DUMM DU M 1 0 -1 1.449 .0 .0 .00000

3 DUMM DU M 2 1 0 1.523 111.21 .0 .00000

4 F4 f M 3 2 1 1.540 111.208 -180.000 -0.100900

5 C14 ca M 4 3 2 1.376 40.370 154.327 0.065400

6 C15 ca S 5 4 3 1.413 119.743 18.427 0.141900

7 F5 f E 6 5 4 1.365 120.513 -1.087 -0.102400

8 C13 ca M 5 4 3 1.391 119.794 -161.514 0.106900

9 F3 f E 8 5 4 1.371 120.367 0.846 -0.096900

10 C12 ca M 8 5 4 1.387 119.220 -179.059 0.065400

11 F2 f E 10 8 5 1.382 114.524 178.667 -0.100900

12 C11 ca M 10 8 5 1.385 121.405 0.409 0.141900

13 F1 f E 12 10 8 1.385 119.874 178.345 -0.102400

14 C10 ca M 12 10 8 1.389 120.179 -1.551 -0.126500

15 C9 cd M 14 12 10 1.478 119.530 -179.115 0.278800

16 N3 nd S 15 14 12 1.405 117.964 -164.357 -0.318000

17 N2 nc E 16 15 14 1.397 103.363 -178.636 -0.147800

18 C8 cc M 15 14 12 1.384 130.839 16.761 -0.178600

19 H11 h4 E 18 15 14 1.072 126.212 -1.744 0.208000

20 N1 na M 18 15 14 1.292 107.575 178.149 0.057400

21 C7 c3 M 20 18 15 1.466 134.754 177.787 0.036000

22 C6 c3 B 21 20 18 1.541 110.362 64.517 0.120100

23 O4 oh S 22 21 20 1.445 108.386 67.472 -0.587800

24 H8 ho E 23 22 21 0.980 109.812 -179.956 0.425000

25 H9 h1 E 22 21 20 1.095 110.695 -54.168 0.075700

26 H10 h1 E 21 20 18 1.090 109.090 -175.540 0.108700

27 C3 c3 M 21 20 18 1.562 109.320 -56.138 0.103100

28 O2 oh S 27 21 20 1.452 109.244 55.366 -0.581800

29 H4 ho E 28 27 21 0.976 110.042 -92.728 0.411000

30 H5 h1 E 27 21 20 1.099 108.207 -62.112 0.064700

31 C2 c3 M 27 21 20 1.545 108.697 -179.280 0.091100

32 C1 c3 3 31 27 21 1.516 110.476 178.911 0.144400

33 O1 oh S 32 31 27 1.453 108.251 174.327 -0.594800

34 H1 ho E 33 32 31 0.977 109.904 -179.980 0.416000

35 H2 h1 E 32 31 27 1.101 109.941 -65.568 0.065700

36 H3 h1 E 32 31 27 1.096 110.130 53.957 0.065700

37 H6 h1 E 31 27 21 1.100 109.476 58.151 0.060700

38 O3 os M 31 27 21 1.460 108.268 -61.302 -0.384600

39 C4 c3 M 38 31 27 1.442 111.310 61.302 0.161200

40 H7 h2 E 39 38 31 1.099 109.023 61.952 0.075700

41 S1 ss M 39 38 31 1.894 108.694 -179.268 -0.181000

42 C5 ca M 41 39 38 1.850 101.074 -63.535 -0.042100

43 C16 ca M 42 41 39 1.402 119.998 -79.520 -0.087500

44 H12 ha E 43 42 41 1.085 119.975 -0.044 0.143000

45 C18 ca M 43 42 41 1.399 120.031 -179.931 -0.136000

46 H14 ha E 45 43 42 1.085 120.020 -179.973 0.139000

47 C20 ca M 45 43 42 1.402 119.935 -0.063 -0.049300

48 C21 c3 3 47 45 43 1.517 119.984 -179.959 -0.060800

49 H16 hc E 48 47 45 1.094 111.268 179.976 0.050033

50 H17 hc E 48 47 45 1.097 111.444 -60.002 0.050033

51 H18 hc E 48 47 45 1.097 111.383 59.978 0.050033

52 C19 ca M 47 45 43 1.402 120.034 0.063 -0.136000

53 H15 ha E 52 47 45 1.085 120.019 -179.935 0.139000

54 C17 ca M 52 47 45 1.399 119.940 -0.054 -0.087500

55 H13 ha E 54 52 47 1.085 119.932 -179.977 0.143000

LOOP

C10 C15

N1 N2

C4 C6

C17 C5

IMPROPER

C15 C13 C14 F4

C10 C14 C15 F5

C14 C12 C13 F3

C11 C13 C12 F2

C10 C12 C11 F1

C11 C15 C10 C9

C10 C8 C9 N3

C9 H11 C8 N1

C7 C8 N1 N2

C16 C17 C5 S1

C5 C18 C16 H12

C16 C20 C18 H14

C21 C18 C20 C19

C20 C17 C19 H15

C5 C19 C17 H13

DONE

STOP

0 0 2

OMe F ligand with BA charges

molecule.res

L1C INT 0

CORRECT OMIT DU BEG

0.0000

1 DUMM DU M 0 -1 -2 0.000 .0 .0 .00000

2 DUMM DU M 1 0 -1 1.449 .0 .0 .00000

3 DUMM DU M 2 1 0 1.523 111.21 .0 .00000

4 F3 f M 3 2 1 1.540 111.208 -180.000 -0.106900

5 C14 ca M 4 3 2 1.384 40.593 151.842 0.083900

6 C15 ca S 5 4 3 1.412 120.037 17.183 0.128900

7 F4 f E 6 5 4 1.368 120.188 -1.184 -0.107400

8 C13 ca M 5 4 3 1.400 120.095 -162.822 0.074100

9 O5 os S 8 5 4 1.371 120.267 0.911 -0.314900

10 C16 c3 3 9 8 5 1.464 127.319 -0.021 0.110700

11 H12 h1 E 10 9 8 1.088 103.589 -179.956 0.052367

12 H13 h1 E 10 9 8 1.091 111.060 -60.006 0.052367

13 H14 h1 E 10 9 8 1.092 111.033 60.055 0.052367

14 C12 ca M 8 5 4 1.385 119.417 -179.070 0.083900

15 F2 f E 14 8 5 1.383 114.307 178.698 -0.106900

16 C11 ca M 14 8 5 1.391 121.740 0.494 0.128900

17 F1 f E 16 14 8 1.385 120.132 178.331 -0.107400

18 C10 ca M 16 14 8 1.394 119.724 -1.664 -0.126500

19 C9 cd M 18 16 14 1.478 119.544 -179.125 0.282800

20 N3 nd S 19 18 16 1.403 117.978 -164.349 -0.320000

21 N2 nc E 20 19 18 1.398 103.390 -178.593 -0.149800

22 C8 cc M 19 18 16 1.386 130.839 16.967 -0.181600

23 H11 h4 E 22 19 18 1.073 126.215 -1.998 0.208000

24 N1 na M 22 19 18 1.292 107.585 178.106 0.055400

25 C7 c3 M 24 22 19 1.465 134.780 177.748 0.036000

26 C6 c3 B 25 24 22 1.542 110.370 64.429 0.120100

27 O4 oh S 26 25 24 1.445 108.393 67.476 -0.587800

28 H8 ho E 27 26 25 0.979 109.819 -179.975 0.424000

29 H9 h1 E 26 25 24 1.095 110.676 -54.171 0.075700

30 H10 h1 E 25 24 22 1.091 109.142 -175.634 0.107700

31 C3 c3 M 25 24 22 1.562 109.340 -56.193 0.103100

32 O2 oh S 31 25 24 1.452 109.216 55.343 -0.582800

33 H4 ho E 32 31 25 0.976 110.036 -92.718 0.412000

34 H5 h1 E 31 25 24 1.100 108.200 -62.097 0.064700

35 C2 c3 M 31 25 24 1.543 108.714 -179.292 0.091100

36 C1 c3 3 35 31 25 1.517 110.491 178.917 0.144400

37 O1 oh S 36 35 31 1.453 108.308 174.306 -0.595800

38 H1 ho E 37 36 35 0.978 109.852 -179.985 0.416000

39 H2 h1 E 36 35 31 1.101 109.907 -65.529 0.065700

40 H3 h1 E 36 35 31 1.095 110.063 53.902 0.065700

41 H6 h1 E 35 31 25 1.100 109.549 58.205 0.060700

42 O3 os M 35 31 25 1.461 108.263 -61.313 -0.384600

43 C4 c3 M 42 35 31 1.441 111.310 61.349 0.161200

44 H7 h2 E 43 42 35 1.098 109.133 61.948 0.075700

45 S1 ss M 43 42 35 1.896 108.679 -179.249 -0.180000

46 C5 ca M 45 43 42 1.850 101.069 -63.560 -0.042100

47 C17 ca M 46 45 43 1.403 119.973 -79.526 -0.088000

48 H15 ha E 47 46 45 1.085 119.991 -0.001 0.143000

49 C19 ca M 47 46 45 1.399 120.004 -179.989 -0.135500

50 H17 ha E 49 47 46 1.086 119.973 179.989 0.138500

51 C21 ca M 49 47 46 1.401 119.978 0.009 -0.049300

52 C22 c3 3 51 49 47 1.516 120.000 -179.977 -0.060800

53 H19 hc E 52 51 49 1.095 111.264 179.998 0.049700

54 H20 hc E 52 51 49 1.097 111.460 -60.039 0.049700

55 H21 hc E 52 51 49 1.097 111.439 59.988 0.049700

56 C20 ca M 51 49 47 1.403 120.018 -0.009 -0.135500

57 H18 ha E 56 51 49 1.085 120.035 -179.965 0.138500

58 C18 ca M 56 51 49 1.399 119.947 -0.005 -0.088000

59 H16 ha E 58 56 51 1.084 119.932 179.982 0.143000

LOOP

C10 C15

N1 N2

C4 C6

C18 C5

IMPROPER

C15 C13 C14 F3

C10 C14 C15 F4

C12 C14 C13 O5

C11 C13 C12 F2

C12 C10 C11 F1

C11 C15 C10 C9

C10 C8 C9 N3

C9 H11 C8 N1

C7 C8 N1 N2

C17 C18 C5 S1

C5 C19 C17 H15

C17 C21 C19 H17

C22 C19 C21 C20

C21 C18 C20 H18

C5 C20 C18 H16

DONE

STOP

0 0 2

MHMe ligand with BA charges

molecule.res

L1d INT 0

CORRECT OMIT DU BEG

0.0000

1 DUMM DU M 0 -1 -2 0.000 .0 .0 .00000

2 DUMM DU M 1 0 -1 1.449 .0 .0 .00000

3 DUMM DU M 2 1 0 1.523 111.21 .0 .00000

4 F3 f M 3 2 1 1.540 111.208 -180.000 -0.125400

5 C14 ca M 4 3 2 1.385 40.877 152.389 0.013400

6 C15 ca S 5 4 3 1.410 120.340 17.058 0.167900

7 F4 f E 6 5 4 1.369 119.921 -1.174 -0.108900

8 C13 ca M 5 4 3 1.401 120.321 -162.964 0.163600

9 N4 nh B 8 5 4 1.380 120.253 0.954 -0.729600

10 H19 hn E 9 8 5 1.008 115.346 -179.945 0.424800

11 C22 c3 3 9 8 5 1.465 129.277 -0.085 0.180100

12 H20 h1 E 11 9 8 1.091 107.430 -179.993 0.050367

13 H21 h1 E 11 9 8 1.094 112.073 -59.910 0.050367

14 H22 h1 E 11 9 8 1.094 111.964 60.027 0.050367

15 C12 ca M 8 5 4 1.391 119.470 -179.065 0.013400

16 F2 f E 15 8 5 1.397 114.151 178.718 -0.125400

17 C11 ca M 15 8 5 1.390 122.031 0.480 0.167900

18 F1 f E 17 15 8 1.386 120.279 178.318 -0.108900

19 C10 ca M 17 15 8 1.393 119.361 -1.581 -0.174500

20 C9 cd M 19 17 15 1.479 119.551 -179.090 0.300800

21 N3 nd S 20 19 17 1.404 117.958 -164.351 -0.325000

22 N2 nc E 21 20 19 1.400 103.320 -178.673 -0.151800

23 C8 cc M 20 19 17 1.386 130.819 16.735 -0.192600

24 H11 h4 E 23 20 19 1.072 126.245 -1.710 0.205000

25 N1 na M 23 20 19 1.294 107.613 178.164 0.053400

26 C7 c3 M 25 23 20 1.464 134.842 177.745 0.037000

27 C6 c3 B 26 25 23 1.542 110.337 64.519 0.120100

28 O4 oh S 27 26 25 1.446 108.377 67.497 -0.586800

29 H8 ho E 28 27 26 0.979 109.785 -179.986 0.423000

30 H9 h1 E 27 26 25 1.095 110.708 -54.094 0.075700

31 H10 h1 E 26 25 23 1.090 109.160 -175.517 0.106700

32 C3 c3 M 26 25 23 1.562 109.309 -56.037 0.104100

33 O2 oh S 32 26 25 1.451 109.282 55.312 -0.582800

34 H4 ho E 33 32 26 0.976 109.971 -92.728 0.412000

35 H5 h1 E 32 26 25 1.101 108.212 -62.189 0.063700

36 C2 c3 M 32 26 25 1.544 108.681 -179.331 0.091100

37 C1 c3 3 36 32 26 1.516 110.500 178.849 0.144400

38 O1 oh S 37 36 32 1.453 108.371 174.325 -0.595800

39 H1 ho E 38 37 36 0.977 109.836 -179.950 0.416000

40 H2 h1 E 37 36 32 1.101 109.934 -65.518 0.065700

41 H3 h1 E 37 36 32 1.096 110.049 53.951 0.065700

42 H6 h1 E 36 32 26 1.101 109.514 58.149 0.060700

43 O3 os M 36 32 26 1.460 108.262 -61.334 -0.385600

44 C4 c3 M 43 36 32 1.441 111.336 61.340 0.162200

45 H7 h2 E 44 43 36 1.099 109.047 61.936 0.075700

46 S1 ss M 44 43 36 1.895 108.702 -179.275 -0.181000

47 C5 ca M 46 44 43 1.849 101.093 -63.573 -0.041100

48 C16 ca M 47 46 44 1.401 120.004 -79.537 -0.088500

49 H12 ha E 48 47 46 1.085 119.973 -0.008 0.143000

50 C18 ca M 48 47 46 1.399 120.018 -179.961 -0.135500

51 H14 ha E 50 48 47 1.087 119.965 -179.936 0.138000

52 C20 ca M 50 48 47 1.401 119.986 -0.041 -0.050300

53 C21 c3 3 52 50 48 1.517 120.017 -179.982 -0.060800

54 H16 hc E 53 52 50 1.094 111.256 179.943 0.049367

55 H17 hc E 53 52 50 1.097 111.496 -59.954 0.049367

56 H18 hc E 53 52 50 1.098 111.401 60.027 0.049367

57 C19 ca M 52 50 48 1.402 119.999 0.080 -0.135500

58 H15 ha E 57 52 50 1.085 120.082 -179.933 0.138000

59 C17 ca M 57 52 50 1.400 119.958 -0.105 -0.088500

60 H13 ha E 59 57 52 1.084 119.929 179.997 0.143000

LOOP

C10 C15

N1 N2

C4 C6

C17 C5

IMPROPER

C15 C13 C14 F3

C10 C14 C15 F4

C12 C14 C13 N4

C22 C13 N4 H19

C11 C13 C12 F2

C12 C10 C11 F1

C11 C15 C10 C9

C10 C8 C9 N3

C9 H11 C8 N1

C7 C8 N1 N2

C16 C17 C5 S1

C5 C18 C16 H12

C16 C20 C18 H14

C21 C18 C20 C19

C20 C17 C19 H15

C5 C19 C17 H13

DONE

STOP

0 0 2

NMe2 ligand with BA charges

molecule.res

L1e INT 0

CORRECT OMIT DU BEG

0.0000

1 DUMM DU M 0 -1 -2 0.000 .0 .0 .00000

2 DUMM DU M 1 0 -1 1.449 .0 .0 .00000

3 DUMM DU M 2 1 0 1.523 111.21 .0 .00000

4 F3 f M 3 2 1 1.540 111.208 -180.000 -0.116900

5 C14 ca M 4 3 2 1.382 41.954 162.428 0.062400

6 C15 ca S 5 4 3 1.420 120.159 16.080 0.135400

7 F4 f E 6 5 4 1.368 120.063 -1.169 -0.110400

8 C13 ca M 5 4 3 1.397 120.196 -163.915 0.122600

9 N4 nh B 8 5 4 1.444 120.229 0.874 -0.643000

10 C22 c3 3 9 8 5 1.480 119.976 -179.967 0.175100

11 H22 h1 E 10 9 8 1.092 107.064 -179.958 0.045700

12 H23 h1 E 10 9 8 1.089 112.562 60.018 0.045700

13 H24 h1 E 10 9 8 1.090 112.370 -60.002 0.045700

14 C23 c3 3 9 8 5 1.474 120.053 0.049 0.175100

15 H19 h1 E 14 9 8 1.091 107.609 179.972 0.045700

16 H20 h1 E 14 9 8 1.092 112.196 -59.977 0.045700

17 H21 h1 E 14 9 8 1.092 112.139 59.917 0.045700

18 C12 ca M 8 5 4 1.406 119.468 -179.059 0.062400

19 F2 f E 18 8 5 1.383 114.257 178.674 -0.116900

20 C11 ca M 18 8 5 1.391 121.798 0.467 0.135400

21 F1 f E 20 18 8 1.387 120.159 178.317 -0.110400

22 C10 ca M 20 18 8 1.396 119.631 -1.598 -0.140500

23 C9 cd M 22 20 18 1.477 119.570 -179.095 0.291800

24 N3 nd S 23 22 20 1.404 117.972 -164.375 -0.321000

25 N2 nc E 24 23 22 1.400 103.313 -178.672 -0.150800

26 C8 cc M 23 22 20 1.385 130.817 16.810 -0.187600

27 H11 h4 E 26 23 22 1.072 126.282 -1.858 0.206000

28 N1 na M 26 23 22 1.294 107.638 178.178 0.053400

29 C7 c3 M 28 26 23 1.464 134.847 177.814 0.037000

30 C6 c3 B 29 28 26 1.542 110.420 64.407 0.120100

31 O4 oh S 30 29 28 1.446 108.372 67.476 -0.586800

32 H8 ho E 31 30 29 0.979 109.859 -179.909 0.424000

33 H9 h1 E 30 29 28 1.095 110.676 -54.101 0.075700

34 H10 h1 E 29 28 26 1.090 109.143 -175.595 0.107700

35 C3 c3 M 29 28 26 1.563 109.297 -56.207 0.103100

36 O2 oh S 35 29 28 1.451 109.251 55.373 -0.582800

37 H4 ho E 36 35 29 0.976 109.946 -92.705 0.412000

38 H5 h1 E 35 29 28 1.100 108.207 -62.121 0.064700

39 C2 c3 M 35 29 28 1.544 108.699 -179.271 0.091100

40 C1 c3 3 39 35 29 1.516 110.476 178.875 0.144400

41 O1 oh S 40 39 35 1.454 108.337 174.282 -0.595800

42 H1 ho E 41 40 39 0.977 109.800 -179.933 0.416000

43 H2 h1 E 40 39 35 1.100 109.988 -65.548 0.065700

44 H3 h1 E 40 39 35 1.096 110.050 53.978 0.065700

45 H6 h1 E 39 35 29 1.100 109.532 58.144 0.060700

46 O3 os M 39 35 29 1.461 108.263 -61.299 -0.385600

47 C4 c3 M 46 39 35 1.441 111.328 61.333 0.162200

48 H7 h2 E 47 46 39 1.098 109.057 61.955 0.075700

49 S1 ss M 47 46 39 1.894 108.694 -179.216 -0.181000

50 C5 ca M 49 47 46 1.850 101.047 -63.571 -0.041100

51 C16 ca M 50 49 47 1.402 119.958 -79.518 -0.088500

52 H12 ha E 51 50 49 1.084 120.006 -0.036 0.143000

53 C18 ca M 51 50 49 1.400 119.951 -179.923 -0.135500

54 H14 ha E 53 51 50 1.086 119.903 -179.962 0.138500

55 C20 ca M 53 51 50 1.401 119.979 -0.069 -0.050300

56 C21 c3 3 55 53 51 1.516 119.979 -179.907 -0.060800

57 H16 hc E 56 55 53 1.095 111.270 179.960 0.049367

58 H17 hc E 56 55 53 1.098 111.447 -60.048 0.049367

59 H18 hc E 56 55 53 1.097 111.447 59.960 0.049367

60 C19 ca M 55 53 51 1.402 120.069 0.069 -0.135500

61 H15 ha E 60 55 53 1.085 120.090 -179.976 0.138500

62 C17 ca M 60 55 53 1.400 119.913 -0.053 -0.088500

63 H13 ha E 62 60 55 1.084 119.923 179.975 0.143000

LOOP

C10 C15

N1 N2

C4 C6

C17 C5

IMPROPER

C15 C13 C14 F3

C10 C14 C15 F4

C12 C14 C13 N4

C23 C22 N4 C13

C11 C13 C12 F2

C12 C10 C11 F1

C11 C15 C10 C9

C10 C8 C9 N3

C9 H11 C8 N1

C7 C8 N1 N2

C16 C17 C5 S1

C5 C18 C16 H12

C16 C20 C18 H14

C21 C18 C20 C19

C20 C17 C19 H15

C5 C19 C17 H13

DONE

STOP

0 0 2

OEt ligand with BA charges

molecule.res

L1G INT 0

CORRECT OMIT DU BEG

0.0000

1 DUMM DU M 0 -1 -2 0.000 .0 .0 .00000

2 DUMM DU M 1 0 -1 1.449 .0 .0 .00000

3 DUMM DU M 2 1 0 1.523 111.21 .0 .00000

4 C17 c3 M 3 2 1 1.540 111.208 -180.000 -0.104100

5 H18 hc E 4 3 2 1.095 149.968 -18.732 0.052700

6 H19 hc E 4 3 2 1.094 77.866 87.804 0.052700

7 H20 hc E 4 3 2 1.093 95.985 -164.916 0.052700

8 C16 c3 M 4 3 2 1.520 43.582 -50.888 0.127400

9 H12 h1 E 8 4 3 1.093 110.787 -136.267 0.052700

10 H13 h1 E 8 4 3 1.093 110.958 103.745 0.052700

11 O5 os M 8 4 3 1.472 105.370 -16.384 -0.313900

12 C13 ca M 11 8 4 1.373 127.744 -179.948 0.104100

13 C14 ca B 12 11 8 1.399 120.286 0.080 0.058400

14 F3 f E 13 12 11 1.383 120.077 0.836 -0.110400

15 C15 ca S 13 12 11 1.412 119.878 -179.106 0.143400

16 F4 f E 15 13 12 1.368 120.220 178.798 -0.106900

17 C12 ca M 12 11 8 1.386 120.261 -179.894 0.058400

18 F2 f E 17 12 11 1.383 114.368 -1.352 -0.110400

19 C11 ca M 17 12 11 1.392 121.685 -179.439 0.143400

20 F1 f E 19 17 12 1.384 120.141 178.284 -0.106900

21 C10 ca M 19 17 12 1.393 119.683 -1.716 -0.144500

22 C9 cd M 21 19 17 1.479 119.512 -179.076 0.288800

23 N3 nd S 22 21 19 1.404 117.972 -164.394 -0.322000

24 N2 nc E 23 22 21 1.398 103.347 -178.634 -0.150800

25 C8 cc M 22 21 19 1.384 130.822 16.873 -0.185600

26 H11 h4 E 25 22 21 1.074 126.244 -1.919 0.207000

27 N1 na M 25 22 21 1.292 107.630 178.127 0.054400

28 C7 c3 M 27 25 22 1.465 134.853 177.782 0.037000

29 C6 c3 B 28 27 25 1.541 110.386 64.397 0.120100

30 O4 oh S 29 28 27 1.445 108.376 67.535 -0.586800

31 H8 ho E 30 29 28 0.979 109.834 -179.956 0.424000

32 H9 h1 E 29 28 27 1.095 110.748 -54.066 0.075700

33 H10 h1 E 28 27 25 1.091 109.176 -175.575 0.107700

34 C3 c3 M 28 27 25 1.563 109.268 -56.204 0.103100

35 O2 oh S 34 28 27 1.452 109.203 55.384 -0.582800

36 H4 ho E 35 34 28 0.976 109.986 -92.808 0.412000

37 H5 h1 E 34 28 27 1.099 108.212 -62.111 0.064700

38 C2 c3 M 34 28 27 1.545 108.654 -179.297 0.091100

39 C1 c3 3 38 34 28 1.516 110.448 178.921 0.144400

40 O1 oh S 39 38 34 1.453 108.274 174.309 -0.595800

41 H1 ho E 40 39 38 0.978 109.791 -179.944 0.416000

42 H2 h1 E 39 38 34 1.101 109.926 -65.506 0.065700

43 H3 h1 E 39 38 34 1.096 110.058 53.905 0.065700

44 H6 h1 E 38 34 28 1.100 109.527 58.164 0.060700

45 O3 os M 38 34 28 1.461 108.236 -61.339 -0.384600

46 C4 c3 M 45 38 34 1.441 111.292 61.417 0.161200

47 H7 h2 E 46 45 38 1.098 109.152 61.882 0.075700

48 S1 ss M 46 45 38 1.896 108.682 -179.301 -0.181000

49 C5 ca M 48 46 45 1.850 101.056 -63.589 -0.041100

50 C18 ca M 49 48 46 1.403 119.960 -79.537 -0.088000

51 H14 ha E 50 49 48 1.085 120.009 0.008 0.143000

52 C20 ca M 50 49 48 1.399 119.942 -179.970 -0.135500

53 H16 ha E 52 50 49 1.086 119.943 -179.999 0.138500

54 C22 ca M 52 50 49 1.401 120.021 0.028 -0.050300

55 C23 c3 3 54 52 50 1.516 120.002 -179.957 -0.060800

56 H21 hc E 55 54 52 1.095 111.255 179.950 0.049367

57 H22 hc E 55 54 52 1.098 111.460 -60.051 0.049367

58 H23 hc E 55 54 52 1.097 111.493 59.932 0.049367

59 C21 ca M 54 52 50 1.402 119.977 -0.055 -0.135500

60 H17 ha E 59 54 52 1.085 120.016 -179.987 0.138500

61 C19 ca M 59 54 52 1.399 120.001 0.055 -0.088000

62 H15 ha E 61 59 54 1.084 119.960 179.949 0.143000

LOOP

C10 C15

N1 N2

C4 C6

C19 C5

IMPROPER

C14 C12 C13 O5

C13 C15 C14 F3

C14 C10 C15 F4

C13 C11 C12 F2

C12 C10 C11 F1

C15 C11 C10 C9

C10 C8 C9 N3

C9 H11 C8 N1

C7 C8 N1 N2

C18 C19 C5 S1

C5 C20 C18 H14

C18 C22 C20 H16

C23 C20 C22 C21

C22 C19 C21 H17

C5 C21 C19 H15

DONE

STOP

0 0 2

Pyr ligand with BA charges

molecule.res

L1l INT 0

CORRECT OMIT DU BEG

0.0000

1 DUMM DU M 0 -1 -2 0.000 .0 .0 .00000

2 DUMM DU M 1 0 -1 1.449 .0 .0 .00000

3 DUMM DU M 2 1 0 1.523 111.21 .0 .00000

4 C21 c3 M 3 2 1 1.540 111.208 -180.000 -0.060800

5 H16 hc E 4 3 2 1.095 71.518 173.751 0.049367

6 H17 hc E 4 3 2 1.098 56.930 47.203 0.049367

7 H18 hc E 4 3 2 1.097 78.116 -72.999 0.049367

8 C20 ca M 4 3 2 1.516 167.638 68.494 -0.051300

9 C19 ca B 8 4 3 1.402 119.991 100.908 -0.135500

10 C17 ca S 9 8 4 1.399 119.978 179.967 -0.088500

11 H13 ha E 10 9 8 1.084 119.917 179.952 0.143500

12 H15 ha E 9 8 4 1.085 120.016 -0.022 0.138000

13 C18 ca M 8 4 3 1.402 120.021 -79.117 -0.135500

14 H14 ha E 13 8 4 1.086 119.988 -0.045 0.138000

15 C16 ca M 13 8 4 1.399 119.960 -179.905 -0.088500

16 H12 ha E 15 13 8 1.085 119.947 -179.938 0.143500

17 C5 ca M 15 13 8 1.402 120.020 -0.125 -0.040100

18 S1 ss M 17 15 13 1.850 119.963 -179.922 -0.182000

19 C4 c3 M 18 17 15 1.895 101.008 -79.494 0.162200

20 H7 h2 E 19 18 17 1.098 109.106 55.309 0.075700

21 C6 c3 B 19 18 17 1.535 106.370 172.746 0.120100

22 O4 oh S 21 19 18 1.446 109.096 -67.068 -0.585800

23 H8 ho E 22 21 19 0.979 109.790 63.636 0.423000

24 H9 h1 E 21 19 18 1.096 110.695 54.978 0.075700

25 O3 os M 19 18 17 1.441 108.724 -63.547 -0.385600

26 C2 c3 M 25 19 18 1.460 111.315 -179.208 0.091100

27 C1 c3 3 26 25 19 1.516 109.406 -178.215 0.144400

28 O1 oh S 27 26 25 1.454 108.372 55.176 -0.596800

29 H1 ho E 28 27 26 0.977 109.821 -179.966 0.416000

30 H2 h1 E 27 26 25 1.101 109.970 175.362 0.065700

31 H3 h1 E 27 26 25 1.096 110.027 -65.167 0.065700

32 H6 h1 E 26 25 19 1.100 109.659 -58.165 0.060700

33 C3 c3 M 26 25 19 1.544 108.305 61.327 0.104100

34 O2 oh S 33 26 25 1.452 114.319 60.988 -0.583800

35 H4 ho E 34 33 26 0.976 109.957 145.217 0.413000

36 H5 h1 E 33 26 25 1.100 108.104 -178.578 0.063700

37 C7 c3 M 33 26 25 1.563 108.670 -61.328 0.038000

38 H10 h1 E 37 33 26 1.090 109.223 -59.933 0.106700

39 N1 na M 37 33 26 1.464 109.308 -179.321 0.052400

40 N2 nc S 39 37 33 1.427 115.214 120.773 -0.152800

41 N3 nd E 40 39 37 1.399 107.959 -178.147 -0.325000

42 C8 cc M 39 37 33 1.294 134.834 -56.121 -0.197600

43 H11 h4 E 42 39 37 1.073 126.129 -2.217 0.204000

44 C9 cd M 42 39 37 1.386 107.611 177.749 0.306800

45 C10 ca M 44 42 39 1.477 130.801 178.152 -0.183500

46 C15 ca B 45 44 42 1.434 120.821 -163.597 0.170400

47 C14 ca S 46 45 44 1.421 120.004 -179.513 0.001900

48 F3 f E 47 46 45 1.382 120.240 178.802 -0.128400

49 F4 f E 46 45 44 1.369 120.003 0.456 -0.110400

50 C11 ca M 45 44 42 1.398 119.585 16.847 0.170400

51 F1 f E 50 45 44 1.386 120.268 0.912 -0.110400

52 C12 ca M 50 45 44 1.391 119.494 -179.078 0.001900

53 F2 f E 52 50 45 1.378 123.901 -179.662 -0.128400

54 C13 ca M 52 50 45 1.410 121.941 -1.653 0.201600

55 N4 nh M 54 52 50 1.463 120.262 -179.458 -0.654000

56 C22 c3 M 55 54 52 1.500 119.887 -0.014 0.199300

57 H19 h1 E 56 55 54 1.066 125.891 -0.062 0.059200

58 H23 h1 E 56 55 54 1.159 90.051 89.963 0.059200

59 C24 c3 M 56 55 54 1.569 101.158 -179.938 -0.089400

60 H22 hc E 59 56 55 1.095 124.444 -179.901 0.056200

61 H26 hc E 59 56 55 1.163 94.388 100.658 0.056200

62 C25 c3 M 59 56 55 1.538 110.598 -0.154 -0.089400

63 H21 hc E 62 59 56 1.097 122.728 -179.966 0.056200

64 H24 hc E 62 59 56 1.137 99.134 -96.479 0.056200

65 C23 c3 M 62 59 56 1.631 107.751 0.159 0.199300

66 H20 h1 E 65 62 59 1.075 133.151 179.894 0.059200

67 H25 h1 E 65 62 59 1.127 89.997 -90.169 0.059200

LOOP

C5 C17

C7 C6

C9 N3

C13 C14

C23 N4

IMPROPER

C21 C18 C20 C19

C20 C17 C19 H15

C5 C19 C17 H13

C16 C20 C18 H14

C18 C5 C16 H12

C16 C17 C5 S1

C7 C8 N1 N2

C9 H11 C8 N1

C10 C8 C9 N3

C15 C11 C10 C9

C10 C14 C15 F4

C15 C13 C14 F3

C10 C12 C11 F1

C11 C13 C12 F2

C14 C12 C13 N4

C22 C23 N4 C13

DONE

STOP

0 0 2

OH ligand with BA charges

molecule.res

L1m INT 0

CORRECT OMIT DU BEG

0.0000

1 DUMM DU M 0 -1 -2 0.000 .0 .0 .00000

2 DUMM DU M 1 0 -1 1.449 .0 .0 .00000

3 DUMM DU M 2 1 0 1.523 111.21 .0 .00000

4 F3 f M 3 2 1 1.540 111.208 -180.000 -0.110900

5 C14 ca M 4 3 2 1.388 40.093 154.209 0.037900

6 C15 ca S 5 4 3 1.416 119.629 18.506 0.159400

7 F4 f E 6 5 4 1.367 120.632 -1.143 -0.104400

8 C13 ca M 5 4 3 1.389 119.632 -161.511 0.112100

9 O5 oh S 8 5 4 1.378 120.240 0.920 -0.463100

10 H19 ho E 9 8 5 0.978 110.552 0.006 0.442000

11 C12 ca M 8 5 4 1.385 119.452 -179.081 0.037900

12 F2 f E 11 8 5 1.381 114.717 178.706 -0.110900

13 C11 ca M 11 8 5 1.389 121.012 0.504 0.159400

14 F1 f E 13 11 8 1.387 119.784 178.307 -0.104400

15 C10 ca M 13 11 8 1.389 120.454 -1.640 -0.153500

16 C9 cd M 15 13 11 1.478 119.582 -179.134 0.287800

17 N3 nd S 16 15 13 1.404 117.982 -164.339 -0.322000

18 N2 nc E 17 16 15 1.398 103.314 -178.633 -0.149800

19 C8 cc M 16 15 13 1.385 130.787 16.912 -0.185600

20 H11 h4 E 19 16 15 1.073 126.258 -1.935 0.207000

21 N1 na M 19 16 15 1.292 107.558 178.147 0.055400

22 C7 c3 M 21 19 16 1.465 134.804 177.805 0.037000

23 C6 c3 B 22 21 19 1.541 110.383 64.352 0.120100

24 O4 oh S 23 22 21 1.445 108.387 67.516 -0.587800

25 H8 ho E 24 23 22 0.979 109.849 -179.982 0.424000

26 H9 h1 E 23 22 21 1.095 110.743 -54.108 0.075700

27 H10 h1 E 22 21 19 1.090 109.172 -175.591 0.107700

28 C3 c3 M 22 21 19 1.563 109.299 -56.211 0.103100

29 O2 oh S 28 22 21 1.452 109.238 55.342 -0.582800

30 H4 ho E 29 28 22 0.977 110.018 -92.728 0.412000

31 H5 h1 E 28 22 21 1.100 108.181 -62.140 0.064700

32 C2 c3 M 28 22 21 1.544 108.713 -179.286 0.091100

33 C1 c3 3 32 28 22 1.516 110.506 178.878 0.144400

34 O1 oh S 33 32 28 1.453 108.328 174.268 -0.595800

35 H1 ho E 34 33 32 0.977 109.908 -179.992 0.416000

36 H2 h1 E 33 32 28 1.100 109.945 -65.543 0.065700

37 H3 h1 E 33 32 28 1.096 110.058 53.933 0.065700

38 H6 h1 E 32 28 22 1.100 109.524 58.115 0.060700

39 O3 os M 32 28 22 1.461 108.251 -61.350 -0.384600

40 C4 c3 M 39 32 28 1.441 111.293 61.378 0.161200

41 H7 h2 E 40 39 32 1.098 109.136 61.931 0.075700

42 S1 ss M 40 39 32 1.894 108.678 -179.255 -0.181000

43 C5 ca M 42 40 39 1.850 101.065 -63.597 -0.041100

44 C16 ca M 43 42 40 1.402 119.961 -79.549 -0.088000

45 H12 ha E 44 43 42 1.085 120.057 0.037 0.143000

46 C18 ca M 44 43 42 1.400 119.974 -179.973 -0.135500

47 H14 ha E 46 44 43 1.086 119.957 -179.929 0.138500

48 C20 ca M 46 44 43 1.402 119.957 0.012 -0.049300

49 C21 c3 3 48 46 44 1.516 119.970 -179.960 -0.060800

50 H16 hc E 49 48 46 1.094 111.273 179.969 0.049700

51 H17 hc E 49 48 46 1.097 111.449 -60.024 0.049700

52 H18 hc E 49 48 46 1.098 111.415 59.978 0.049700

53 C19 ca M 48 46 44 1.402 119.991 0.024 -0.135500

54 H15 ha E 53 48 46 1.086 119.996 -179.967 0.138500

55 C17 ca M 53 48 46 1.399 120.031 -0.051 -0.088000

56 H13 ha E 55 53 48 1.084 119.996 179.953 0.143000

LOOP

C10 C15

N1 N2

C4 C6

C17 C5

IMPROPER

C15 C13 C14 F3

C10 C14 C15 F4

C12 C14 C13 O5

C11 C13 C12 F2

C12 C10 C11 F1

C11 C15 C10 C9

C10 C8 C9 N3

C9 H11 C8 N1

C7 C8 N1 N2

C16 C17 C5 S1

C5 C18 C16 H12

C16 C20 C18 H14

C21 C18 C20 C19

C20 C17 C19 H15

C5 C19 C17 H13

DONE

STOP

0 0 2

NH2 ligand with BA charges

molecule.res

L1n INT 0

CORRECT OMIT DU BEG

0.0000

1 DUMM DU M 0 -1 -2 0.000 .0 .0 .00000

2 DUMM DU M 1 0 -1 1.449 .0 .0 .00000

3 DUMM DU M 2 1 0 1.523 111.21 .0 .00000

4 F3 f M 3 2 1 1.540 111.208 -180.000 -0.124900

5 C14 ca M 4 3 2 1.386 40.480 154.660 0.008900

6 C15 ca S 5 4 3 1.414 120.010 18.346 0.171900

7 F4 f E 6 5 4 1.368 120.273 -1.109 -0.108400

8 C13 ca M 5 4 3 1.393 119.978 -161.630 0.159600

9 N4 nh B 8 5 4 1.375 120.227 0.910 -0.821200

10 H19 hn E 9 8 5 1.004 120.152 179.995 0.425300

11 H20 hn E 9 8 5 1.004 120.073 -0.029 0.425300

12 C12 ca M 8 5 4 1.390 119.479 -179.117 0.008900

13 F2 f E 12 8 5 1.394 114.461 178.677 -0.124900

14 C11 ca M 12 8 5 1.388 121.541 0.544 0.171900

15 F1 f E 14 12 8 1.387 120.051 178.299 -0.108400

16 C10 ca M 14 12 8 1.391 119.872 -1.672 -0.175500

17 C9 cd M 16 14 12 1.479 119.551 -179.114 0.298800

18 N3 nd S 17 16 14 1.403 118.004 -164.377 -0.325000

19 N2 nc E 18 17 16 1.399 103.342 -178.601 -0.151800

20 C8 cc M 17 16 14 1.387 130.803 16.897 -0.192600

21 H11 h4 E 20 17 16 1.072 126.251 -1.926 0.205000

22 N1 na M 20 17 16 1.293 107.552 178.103 0.053400

23 C7 c3 M 22 20 17 1.464 134.793 177.746 0.037000

24 C6 c3 B 23 22 20 1.542 110.362 64.468 0.120100

25 O4 oh S 24 23 22 1.445 108.401 67.506 -0.586800

26 H8 ho E 25 24 23 0.980 109.815 -179.965 0.423000

27 H9 h1 E 24 23 22 1.095 110.710 -54.144 0.075700

28 H10 h1 E 23 22 20 1.091 109.218 -175.553 0.106700

29 C3 c3 M 23 22 20 1.563 109.315 -56.119 0.104100

30 O2 oh S 29 23 22 1.451 109.265 55.316 -0.582800

31 H4 ho E 30 29 23 0.976 109.966 -92.691 0.412000

32 H5 h1 E 29 23 22 1.100 108.200 -62.201 0.063700

33 C2 c3 M 29 23 22 1.545 108.692 -179.284 0.091100

34 C1 c3 3 33 29 23 1.517 110.471 178.896 0.144400

35 O1 oh S 34 33 29 1.453 108.317 174.289 -0.595800

36 H1 ho E 35 34 33 0.977 109.809 179.989 0.416000

37 H2 h1 E 34 33 29 1.101 109.899 -65.581 0.065700

38 H3 h1 E 34 33 29 1.096 110.065 53.902 0.065700

39 H6 h1 E 33 29 23 1.100 109.548 58.132 0.060700

40 O3 os M 33 29 23 1.461 108.231 -61.385 -0.385600

41 C4 c3 M 40 33 29 1.441 111.328 61.401 0.162200

42 H7 h2 E 41 40 33 1.099 109.116 61.848 0.075700

43 S1 ss M 41 40 33 1.895 108.716 -179.249 -0.181000

44 C5 ca M 43 41 40 1.850 101.030 -63.622 -0.041100

45 C16 ca M 44 43 41 1.402 119.989 -79.539 -0.088500

46 H12 ha E 45 44 43 1.084 120.042 0.012 0.143000

47 C18 ca M 45 44 43 1.400 119.956 -179.977 -0.135500

48 H14 ha E 47 45 44 1.086 119.928 -179.981 0.138500

49 C20 ca M 47 45 44 1.402 119.986 0.035 -0.050300

50 C21 c3 3 49 47 45 1.517 119.974 -179.992 -0.060800

51 H16 hc E 50 49 47 1.094 111.244 -179.999 0.049367

52 H17 hc E 50 49 47 1.098 111.433 -60.031 0.049367

53 H18 hc E 50 49 47 1.097 111.424 59.969 0.049367

54 C19 ca M 49 47 45 1.402 120.002 -0.024 -0.135500

55 H15 ha E 54 49 47 1.085 120.009 179.996 0.138500

56 C17 ca M 54 49 47 1.399 119.957 0.020 -0.088500

57 H13 ha E 56 54 49 1.085 119.929 179.932 0.143000

LOOP

C10 C15

N1 N2

C4 C6

C17 C5

IMPROPER

C15 C13 C14 F3

C10 C14 C15 F4

C12 C14 C13 N4

C13 H20 N4 H19

C11 C13 C12 F2

C12 C10 C11 F1

C11 C15 C10 C9

C10 C8 C9 N3

C9 H11 C8 N1

C7 C8 N1 N2

C16 C17 C5 S1

C5 C18 C16 H12

C16 C20 C18 H14

C21 C18 C20 C19

C20 C17 C19 H15

C5 C19 C17 H13

DONE

STOP

BH charges

0 0 2

F ligand with BH charges

molecule.res

L1b INT 0

CORRECT OMIT DU BEG

0.0000

1 DUMM DU M 0 -1 -2 0.000 .0 .0 .00000

2 DUMM DU M 1 0 -1 1.449 .0 .0 .00000

3 DUMM DU M 2 1 0 1.523 111.21 .0 .00000

4 F4 f M 3 2 1 1.540 111.208 -180.000 -0.101900

5 C14 ca M 4 3 2 1.374 141.140 46.175 0.064900

6 C15 ca S 5 4 3 1.393 119.955 157.514 0.141400

7 F5 f E 6 5 4 1.374 116.776 0.761 -0.101900

8 C13 ca M 5 4 3 1.391 119.589 -22.946 0.105900

9 F3 f E 8 5 4 1.372 120.463 0.337 -0.097900

10 C12 ca M 8 5 4 1.390 119.165 -179.470 0.064900

11 F2 f E 10 8 5 1.375 120.110 179.951 -0.101900

12 C11 ca M 10 8 5 1.389 119.614 -0.207 0.141400

13 F1 f E 12 10 8 1.385 117.276 -179.859 -0.101900

14 C10 ca M 12 10 8 1.405 123.111 -0.055 -0.123500

15 C9 cd M 14 12 10 1.460 121.105 -179.631 0.263800

16 N3 nd S 15 14 12 1.388 122.286 163.734 -0.310000

17 N2 nc E 16 15 14 1.315 109.112 -179.763 -0.188800

18 C8 cc M 15 14 12 1.394 129.509 -15.849 -0.141600

19 H11 h4 E 18 15 14 1.073 132.385 -2.710 0.222000

20 N1 na M 18 15 14 1.358 105.329 179.703 0.053400

21 C7 c3 M 20 18 15 1.473 128.497 179.554 0.039000

22 C6 c3 B 21 20 18 1.538 111.380 -70.848 0.119100

23 O4 oh S 22 21 20 1.448 106.047 60.387 -0.609800

24 H8 ho E 23 22 21 0.980 109.793 178.512 0.430000

25 H9 h1 E 22 21 20 1.097 109.416 -60.631 0.094700

26 H10 h1 E 21 20 18 1.098 106.486 45.924 0.080700

27 C3 c3 M 21 20 18 1.549 111.540 164.196 0.127100

28 O2 oh S 27 21 20 1.442 111.772 60.650 -0.581800

29 H4 ho E 28 27 21 0.987 107.703 -63.795 0.428000

30 H5 h1 E 27 21 20 1.100 108.341 -61.825 0.069700

31 C2 c3 M 27 21 20 1.532 109.342 179.912 0.088100

32 C1 c3 3 31 27 21 1.524 114.062 -172.043 0.143400

33 O1 oh S 32 31 27 1.450 110.298 178.705 -0.595800

34 H1 ho E 33 32 31 0.980 107.813 -65.744 0.415000

35 H2 h1 E 32 31 27 1.092 110.210 -64.418 0.068200

36 H3 h1 E 32 31 27 1.093 108.960 55.272 0.068200

37 H6 h1 E 31 27 21 1.101 109.543 64.630 0.056700

38 O3 os M 31 27 21 1.476 110.569 -54.610 -0.390600

39 C4 c3 M 38 31 27 1.442 113.463 58.855 0.162200

40 H7 h2 E 39 38 31 1.099 111.174 62.626 0.075700

41 S1 ss M 39 38 31 1.886 108.188 -179.992 -0.166000

42 C5 ca M 41 39 38 1.855 100.404 -89.660 -0.027100

43 C16 ca M 42 41 39 1.399 118.660 -135.793 -0.108000

44 H12 ha E 43 42 41 1.084 120.213 2.784 0.142500

45 C18 ca M 43 42 41 1.398 119.641 -177.693 -0.124500

46 H14 ha E 45 43 42 1.086 119.294 -179.880 0.137500

47 C20 ca M 45 43 42 1.406 121.222 -0.766 -0.065300

48 C21 c3 3 47 45 43 1.512 121.003 179.138 -0.057800

49 H16 hc E 48 47 45 1.095 111.517 148.284 0.048033

50 H17 hc E 48 47 45 1.099 111.174 -92.152 0.048033

51 H18 hc E 48 47 45 1.096 111.467 27.480 0.048033

52 C19 ca M 47 45 43 1.406 118.079 0.245 -0.124500

53 H15 ha E 52 47 45 1.086 119.525 -179.832 0.137500

54 C17 ca M 52 47 45 1.399 121.354 0.861 -0.108000

55 H13 ha E 54 52 47 1.084 120.220 178.810 0.142500

LOOP

C10 C15

N1 N2

C4 C6

C17 C5

IMPROPER

C15 C13 C14 F4

C10 C14 C15 F5

C14 C12 C13 F3

C11 C13 C12 F2

C10 C12 C11 F1

C11 C15 C10 C9

C10 C8 C9 N3

C9 H11 C8 N1

C7 C8 N1 N2

C16 C17 C5 S1

C5 C18 C16 H12

C16 C20 C18 H14

C21 C18 C20 C19

C20 C17 C19 H15

C5 C19 C17 H13

DONE

STOP

0 0 2

OMe ligand with BH charges

molecule.res

L1C INT 0

CORRECT OMIT DU BEG

0.0000

1 DUMM DU M 0 -1 -2 0.000 .0 .0 .00000

2 DUMM DU M 1 0 -1 1.449 .0 .0 .00000

3 DUMM DU M 2 1 0 1.523 111.21 .0 .00000

4 F3 f M 3 2 1 1.540 111.208 -180.000 -0.110900

5 C14 ca M 4 3 2 1.390 138.736 43.144 0.056900

6 C15 ca S 5 4 3 1.394 117.623 161.034 0.144900

7 F4 f E 6 5 4 1.375 116.537 1.048 -0.106400

8 C13 ca M 5 4 3 1.399 120.323 -19.266 0.101100

9 O5 os S 8 5 4 1.368 127.903 0.696 -0.306900

10 C16 c3 3 9 8 5 1.470 121.983 -11.254 0.109700

11 H12 h1 E 10 9 8 1.088 103.711 -173.727 0.056367

12 H13 h1 E 10 9 8 1.093 110.743 -55.155 0.056367

13 H14 h1 E 10 9 8 1.091 110.911 67.461 0.056367

14 C12 ca M 8 5 4 1.405 116.092 179.704 0.056900

15 F2 f E 14 8 5 1.377 119.248 179.972 -0.110900

16 C11 ca M 14 8 5 1.384 121.297 0.105 0.144900

17 F1 f E 16 14 8 1.388 117.260 179.934 -0.106400

18 C10 ca M 16 14 8 1.406 123.420 0.088 -0.141500

19 C9 cd M 18 16 14 1.459 121.527 179.990 0.272800

20 N3 nd S 19 18 16 1.388 122.450 164.907 -0.314000

21 N2 nc E 20 19 18 1.317 109.098 -179.805 -0.191800

22 C8 cc M 19 18 16 1.394 129.401 -14.600 -0.146600

23 H11 h4 E 22 19 18 1.073 132.256 -2.730 0.221000

24 N1 na M 22 19 18 1.359 105.423 179.687 0.051400

25 C7 c3 M 24 22 19 1.472 128.517 179.347 0.039000

26 C6 c3 B 25 24 22 1.538 111.423 -72.440 0.119100

27 O4 oh S 26 25 24 1.449 105.962 60.643 -0.608800

28 H8 ho E 27 26 25 0.980 109.781 179.308 0.430000

29 H9 h1 E 26 25 24 1.095 109.443 -60.416 0.093700

30 H10 h1 E 25 24 22 1.097 106.488 44.399 0.079700

31 C3 c3 M 25 24 22 1.549 111.482 162.639 0.127100

32 O2 oh S 31 25 24 1.441 111.891 60.607 -0.582800

33 H4 ho E 32 31 25 0.988 107.536 -62.388 0.428000

34 H5 h1 E 31 25 24 1.101 108.293 -61.874 0.069700

35 C2 c3 M 31 25 24 1.532 109.340 179.944 0.088100

36 C1 c3 3 35 31 25 1.525 114.062 -172.012 0.142400

37 O1 oh S 36 35 31 1.451 110.329 178.298 -0.596800

38 H1 ho E 37 36 35 0.979 107.643 -65.217 0.415000

39 H2 h1 E 36 35 31 1.092 110.204 -64.785 0.067700

40 H3 h1 E 36 35 31 1.093 108.948 54.934 0.067700

41 H6 h1 E 35 31 25 1.101 109.591 64.586 0.055700

42 O3 os M 35 31 25 1.476 110.550 -54.687 -0.391600

43 C4 c3 M 42 35 31 1.443 113.429 58.897 0.163200

44 H7 h2 E 43 42 35 1.098 111.178 62.630 0.075700

45 S1 ss M 43 42 35 1.887 108.142 179.908 -0.166000

46 C5 ca M 45 43 42 1.853 100.389 -89.484 -0.026100

47 C17 ca M 46 45 43 1.399 118.652 -135.953 -0.108500

48 H15 ha E 47 46 45 1.084 120.176 2.751 0.142500

49 C19 ca M 47 46 45 1.399 119.650 -177.802 -0.124000

50 H17 ha E 49 47 46 1.086 119.289 -179.945 0.137500

51 C21 ca M 49 47 46 1.405 121.213 -0.742 -0.066300

52 C22 c3 3 51 49 47 1.513 120.999 179.119 -0.057800

53 H19 hc E 52 51 49 1.095 111.459 148.113 0.047700

54 H20 hc E 52 51 49 1.098 111.174 -92.337 0.047700

55 H21 hc E 52 51 49 1.095 111.435 27.356 0.047700

56 C20 ca M 51 49 47 1.406 118.103 0.179 -0.124000

57 H18 ha E 56 51 49 1.086 119.530 -179.854 0.137500

58 C18 ca M 56 51 49 1.398 121.340 0.895 -0.108500

59 H16 ha E 58 56 51 1.085 120.283 178.783 0.142500

LOOP

C10 C15

N1 N2

C4 C6

C18 C5

IMPROPER

C15 C13 C14 F3

C10 C14 C15 F4

C12 C14 C13 O5

C11 C13 C12 F2

C12 C10 C11 F1

C11 C15 C10 C9

C10 C8 C9 N3

C9 H11 C8 N1

C7 C8 N1 N2

C17 C18 C5 S1

C5 C19 C17 H15

C17 C21 C19 H17

C22 C19 C21 C20

C21 C18 C20 H18

C5 C20 C18 H16

DONE

STOP

0 0 2

NHMe ligand with BH charges

molecule.res

L1d INT 0

CORRECT OMIT DU BEG

0.0000

1 DUMM DU M 0 -1 -2 0.000 .0 .0 .00000

2 DUMM DU M 1 0 -1 1.449 .0 .0 .00000

3 DUMM DU M 2 1 0 1.523 111.21 .0 .00000

4 F3 f M 3 2 1 1.540 111.208 -180.000 -0.125400

5 C14 ca M 4 3 2 1.391 138.183 44.825 0.013900

6 C15 ca S 5 4 3 1.392 117.892 160.447 0.166900

7 F4 f E 6 5 4 1.376 116.448 0.698 -0.108400

8 C13 ca M 5 4 3 1.406 119.650 -20.044 0.160600

9 N4 nh B 8 5 4 1.370 126.327 0.414 -0.728600

10 H19 hn E 9 8 5 1.007 115.442 179.803 0.423800

11 C22 c3 3 9 8 5 1.464 126.664 -0.084 0.180100

12 H20 h1 E 11 9 8 1.092 107.611 -179.913 0.049700

13 H21 h1 E 11 9 8 1.094 111.865 -60.719 0.049700

14 H22 h1 E 11 9 8 1.094 111.859 60.996 0.049700

15 C12 ca M 8 5 4 1.410 114.505 -179.441 0.013900

16 F2 f E 15 8 5 1.394 117.168 179.912 -0.125400

17 C11 ca M 15 8 5 1.380 122.782 -0.286 0.166900

18 F1 f E 17 15 8 1.390 117.478 -179.812 -0.108400

19 C10 ca M 17 15 8 1.406 123.010 0.080 -0.170500

20 C9 cd M 19 17 15 1.459 121.669 -179.697 0.284800

21 N3 nd S 20 19 17 1.388 122.540 164.242 -0.316000

22 N2 nc E 21 20 19 1.318 109.112 -179.803 -0.193800

23 C8 cc M 20 19 17 1.395 129.372 -15.247 -0.153600

24 H11 h4 E 23 20 19 1.072 132.182 -2.748 0.219000

25 N1 na M 23 20 19 1.359 105.433 179.563 0.049400

26 C7 c3 M 25 23 20 1.472 128.563 179.415 0.040000

27 C6 c3 B 26 25 23 1.538 111.514 -72.938 0.119100

28 O4 oh S 27 26 25 1.448 105.992 60.750 -0.607800

29 H8 ho E 28 27 26 0.980 109.791 -179.986 0.429000

30 H9 h1 E 27 26 25 1.096 109.331 -60.308 0.093700

31 H10 h1 E 26 25 23 1.098 106.473 43.820 0.079700

32 C3 c3 M 26 25 23 1.549 111.479 162.035 0.127100

33 O2 oh S 32 26 25 1.441 111.881 60.613 -0.582800

34 H4 ho E 33 32 26 0.989 107.426 -61.722 0.429000

35 H5 h1 E 32 26 25 1.101 108.234 -61.875 0.068700

36 C2 c3 M 32 26 25 1.532 109.417 179.989 0.088100

37 C1 c3 3 36 32 26 1.524 114.061 -171.976 0.142400

38 O1 oh S 37 36 32 1.451 110.328 178.141 -0.596800

39 H1 ho E 38 37 36 0.979 107.663 -65.042 0.415000

40 H2 h1 E 37 36 32 1.093 110.260 -64.948 0.067700

41 H3 h1 E 37 36 32 1.093 108.952 54.805 0.067700

42 H6 h1 E 36 32 26 1.100 109.538 64.556 0.055700

43 O3 os M 36 32 26 1.476 110.536 -54.635 -0.391600

44 C4 c3 M 43 36 32 1.443 113.482 58.786 0.163200

45 H7 h2 E 44 43 36 1.098 111.100 62.731 0.074700

46 S1 ss M 44 43 36 1.887 108.118 179.968 -0.166000

47 C5 ca M 46 44 43 1.853 100.432 -89.184 -0.025100

48 C16 ca M 47 46 44 1.399 118.690 -136.390 -0.109000

49 H12 ha E 48 47 46 1.085 120.131 2.803 0.142500

50 C18 ca M 48 47 46 1.399 119.701 -177.764 -0.124000

51 H14 ha E 50 48 47 1.086 119.273 -179.904 0.137000

52 C20 ca M 50 48 47 1.405 121.221 -0.728 -0.066300

53 C21 c3 3 52 50 48 1.513 121.029 179.114 -0.056800

54 H16 hc E 53 52 50 1.095 111.498 148.334 0.047700

55 H17 hc E 53 52 50 1.099 111.168 -92.026 0.047700

56 H18 hc E 53 52 50 1.096 111.423 27.536 0.047700

57 C19 ca M 52 50 48 1.406 118.082 0.204 -0.124000

58 H15 ha E 57 52 50 1.086 119.533 -179.848 0.137000

59 C17 ca M 57 52 50 1.398 121.338 0.883 -0.109000

60 H13 ha E 59 57 52 1.085 120.257 178.806 0.142500

LOOP

C10 C15

N1 N2

C4 C6

C17 C5

IMPROPER

C15 C13 C14 F3

C10 C14 C15 F4

C12 C14 C13 N4

C22 C13 N4 H19

C11 C13 C12 F2

C12 C10 C11 F1

C11 C15 C10 C9

C10 C8 C9 N3

C9 H11 C8 N1

C7 C8 N1 N2

C16 C17 C5 S1

C5 C18 C16 H12

C16 C20 C18 H14

C21 C18 C20 C19

C20 C17 C19 H15

C5 C19 C17 H13

DONE

STOP

0 0 2

NMe2 ligand with BH charges

molecule.res

L1e INT 0

CORRECT OMIT DU BEG

0.0000

1 DUMM DU M 0 -1 -2 0.000 .0 .0 .00000

2 DUMM DU M 1 0 -1 1.449 .0 .0 .00000

3 DUMM DU M 2 1 0 1.523 111.21 .0 .00000

4 F3 f M 3 2 1 1.540 111.208 -180.000 -0.117900

5 C14 ca M 4 3 2 1.388 138.135 36.796 0.062900

6 C15 ca S 5 4 3 1.389 117.665 164.409 0.134400

7 F4 f E 6 5 4 1.377 116.714 2.175 -0.109900

8 C13 ca M 5 4 3 1.413 119.055 -14.501 0.119600

9 N4 nh B 8 5 4 1.382 123.070 -2.262 -0.642000

10 C22 c3 3 9 8 5 1.468 122.277 145.596 0.174100

11 H22 h1 E 10 9 8 1.097 108.699 -135.002 0.045533

12 H23 h1 E 10 9 8 1.097 112.061 105.049 0.045533

13 H24 h1 E 10 9 8 1.089 110.689 -16.224 0.045533

14 C23 c3 3 9 8 5 1.468 122.383 -34.615 0.174100

15 H19 h1 E 14 9 8 1.098 108.753 -134.208 0.045533

16 H20 h1 E 14 9 8 1.089 110.697 -15.335 0.045533

17 H21 h1 E 14 9 8 1.097 111.969 105.813 0.045533

18 C12 ca M 8 5 4 1.411 113.913 177.920 0.062900

19 F2 f E 18 8 5 1.390 119.509 177.337 -0.117900

20 C11 ca M 18 8 5 1.385 122.478 -1.140 0.134400

21 F1 f E 20 18 8 1.390 117.131 -179.960 -0.109900

22 C10 ca M 20 18 8 1.404 123.562 2.052 -0.136500

23 C9 cd M 22 20 18 1.459 121.752 179.121 0.275800

24 N3 nd S 23 22 20 1.388 122.516 165.650 -0.313000

25 N2 nc E 24 23 22 1.318 109.122 -179.989 -0.192800

26 C8 cc M 23 22 20 1.394 129.377 -14.061 -0.148600

27 H11 h4 E 26 23 22 1.072 132.209 -2.577 0.221000

28 N1 na M 26 23 22 1.359 105.451 179.801 0.050400

29 C7 c3 M 28 26 23 1.471 128.569 179.397 0.040000

30 C6 c3 B 29 28 26 1.538 111.459 -72.437 0.119100

31 O4 oh S 30 29 28 1.448 106.049 60.652 -0.607800

32 H8 ho E 31 30 29 0.979 109.881 179.813 0.429000

33 H9 h1 E 30 29 28 1.096 109.399 -60.389 0.093700

34 H10 h1 E 29 28 26 1.097 106.503 44.322 0.079700

35 C3 c3 M 29 28 26 1.550 111.485 162.615 0.127100

36 O2 oh S 35 29 28 1.441 111.841 60.516 -0.582800

37 H4 ho E 36 35 29 0.988 107.438 -61.989 0.428000

38 H5 h1 E 35 29 28 1.101 108.208 -61.921 0.069700

39 C2 c3 M 35 29 28 1.532 109.371 179.897 0.088100

40 C1 c3 3 39 35 29 1.524 114.046 -171.972 0.142400

41 O1 oh S 40 39 35 1.451 110.378 178.109 -0.596800

42 H1 ho E 41 40 39 0.979 107.656 -65.141 0.415000

43 H2 h1 E 40 39 35 1.093 110.271 -64.921 0.067700

44 H3 h1 E 40 39 35 1.093 108.908 54.752 0.067700

45 H6 h1 E 39 35 29 1.102 109.545 64.632 0.055700

46 O3 os M 39 35 29 1.476 110.588 -54.583 -0.391600

47 C4 c3 M 46 39 35 1.442 113.466 58.817 0.163200

48 H7 h2 E 47 46 39 1.098 111.203 62.692 0.075700

49 S1 ss M 47 46 39 1.888 108.101 179.863 -0.166000

50 C5 ca M 49 47 46 1.853 100.323 -89.263 -0.025100

51 C16 ca M 50 49 47 1.399 118.668 -136.219 -0.109000

52 H12 ha E 51 50 49 1.084 120.143 2.854 0.142500

53 C18 ca M 51 50 49 1.399 119.641 -177.818 -0.124000

54 H14 ha E 53 51 50 1.087 119.250 -179.793 0.137500

55 C20 ca M 53 51 50 1.405 121.213 -0.740 -0.066300

56 C21 c3 3 55 53 51 1.513 120.973 179.139 -0.057800

57 H16 hc E 56 55 53 1.095 111.522 148.697 0.047700

58 H17 hc E 56 55 53 1.099 111.191 -91.731 0.047700

59 H18 hc E 56 55 53 1.095 111.487 27.877 0.047700

60 C19 ca M 55 53 51 1.405 118.122 0.229 -0.124000

61 H15 ha E 60 55 53 1.086 119.530 -179.931 0.137500

62 C17 ca M 60 55 53 1.398 121.318 0.883 -0.109000

63 H13 ha E 62 60 55 1.084 120.220 178.774 0.142500

LOOP

C10 C15

N1 N2

C4 C6

C17 C5

IMPROPER

C15 C13 C14 F3

C10 C14 C15 F4

C12 C14 C13 N4

C23 C22 N4 C13

C11 C13 C12 F2

C12 C10 C11 F1

C11 C15 C10 C9

C10 C8 C9 N3

C9 H11 C8 N1

C7 C8 N1 N2

C16 C17 C5 S1

C5 C18 C16 H12

C16 C20 C18 H14

C21 C18 C20 C19

C20 C17 C19 H15

C5 C19 C17 H13

DONE

STOP

0 0 2

OEt ligand with BH charges

molecule.res

L1G INT 0

CORRECT OMIT DU BEG

0.0000

1 DUMM DU M 0 -1 -2 0.000 .0 .0 .00000

2 DUMM DU M 1 0 -1 1.449 .0 .0 .00000

3 DUMM DU M 2 1 0 1.523 111.21 .0 .00000

4 C17 c3 M 3 2 1 1.540 111.208 -180.000 -0.104100

5 H18 hc E 4 3 2 1.096 25.123 -157.901 0.052367

6 H19 hc E 4 3 2 1.093 94.721 76.576 0.052367

7 H20 hc E 4 3 2 1.094 94.509 -32.378 0.052367

8 C16 c3 M 4 3 2 1.517 134.875 -157.515 0.127400

9 H12 h1 E 8 4 3 1.092 112.348 60.268 0.052200

10 H13 h1 E 8 4 3 1.095 112.271 -63.019 0.052200

11 O5 os M 8 4 3 1.486 105.585 178.702 -0.314900

12 C13 ca M 11 8 4 1.368 122.019 168.417 0.103100

13 C14 ca B 12 11 8 1.400 127.375 20.307 0.058400

14 F3 f E 13 12 11 1.389 120.164 -0.311 -0.110900

15 C15 ca S 13 12 11 1.394 122.043 178.838 0.142900

16 F4 f E 15 13 12 1.377 116.588 -179.042 -0.106400

17 C12 ca M 12 11 8 1.405 116.501 -161.776 0.058400

18 F2 f E 17 12 11 1.377 119.263 1.790 -0.110900

19 C11 ca M 17 12 11 1.384 121.309 -178.725 0.142900

20 F1 f E 19 17 12 1.388 117.329 -179.675 -0.106400

21 C10 ca M 19 17 12 1.406 123.431 -0.240 -0.140500

22 C9 cd M 21 19 17 1.459 121.562 -179.490 0.272800

23 N3 nd S 22 21 19 1.388 122.410 164.176 -0.314000

24 N2 nc E 23 22 21 1.318 109.060 -179.783 -0.191800

25 C8 cc M 22 21 19 1.394 129.405 -15.315 -0.146600

26 H11 h4 E 25 22 21 1.072 132.252 -2.745 0.221000

27 N1 na M 25 22 21 1.359 105.382 179.652 0.051400

28 C7 c3 M 27 25 22 1.472 128.492 179.292 0.040000

29 C6 c3 B 28 27 25 1.538 111.353 -72.479 0.119100

30 O4 oh S 29 28 27 1.448 105.983 60.561 -0.608800

31 H8 ho E 30 29 28 0.979 109.865 179.457 0.430000

32 H9 h1 E 29 28 27 1.095 109.505 -60.557 0.093700

33 H10 h1 E 28 27 25 1.097 106.494 44.293 0.079700

34 C3 c3 M 28 27 25 1.549 111.558 162.637 0.127100

35 O2 oh S 34 28 27 1.442 111.864 60.564 -0.582800

36 H4 ho E 35 34 28 0.988 107.483 -62.215 0.428000

37 H5 h1 E 34 28 27 1.101 108.263 -61.887 0.069700

38 C2 c3 M 34 28 27 1.532 109.353 179.898 0.088100

39 C1 c3 3 38 34 28 1.524 114.025 -172.031 0.142400

40 O1 oh S 39 38 34 1.451 110.328 178.279 -0.596800

41 H1 ho E 40 39 38 0.980 107.690 -65.278 0.415000

42 H2 h1 E 39 38 34 1.093 110.308 -64.811 0.067700

43 H3 h1 E 39 38 34 1.093 108.925 54.908 0.067700

44 H6 h1 E 38 34 28 1.101 109.491 64.640 0.055700

45 O3 os M 38 34 28 1.475 110.604 -54.649 -0.391600

46 C4 c3 M 45 38 34 1.443 113.470 58.820 0.163200

47 H7 h2 E 46 45 38 1.098 111.168 62.707 0.075700

48 S1 ss M 46 45 38 1.886 108.150 179.924 -0.166000

49 C5 ca M 48 46 45 1.854 100.377 -89.377 -0.026100

50 C18 ca M 49 48 46 1.398 118.679 -136.013 -0.108500

51 H14 ha E 50 49 48 1.085 120.164 2.665 0.142500

52 C20 ca M 50 49 48 1.399 119.693 -177.844 -0.124000

53 H16 ha E 52 50 49 1.086 119.289 -179.940 0.137500

54 C22 ca M 52 50 49 1.405 121.166 -0.775 -0.066300

55 C23 c3 3 54 52 50 1.513 120.970 179.153 -0.057800

56 H21 hc E 55 54 52 1.095 111.478 148.489 0.047700

57 H22 hc E 55 54 52 1.098 111.173 -92.001 0.047700

58 H23 hc E 55 54 52 1.095 111.485 27.609 0.047700

59 C21 ca M 54 52 50 1.405 118.113 0.201 -0.124000

60 H17 ha E 59 54 52 1.087 119.477 -179.865 0.137500

61 C19 ca M 59 54 52 1.399 121.369 0.881 -0.108500

62 H15 ha E 61 59 54 1.084 120.245 178.757 0.142500

LOOP

C10 C15

N1 N2

C4 C6

C19 C5

IMPROPER

C14 C12 C13 O5

C13 C15 C14 F3

C14 C10 C15 F4

C13 C11 C12 F2

C12 C10 C11 F1

C15 C11 C10 C9

C10 C8 C9 N3

C9 H11 C8 N1

C7 C8 N1 N2

C18 C19 C5 S1

C5 C20 C18 H14

C18 C22 C20 H16

C23 C20 C22 C21

C22 C19 C21 H17

C5 C21 C19 H15

DONE

STOP

0 0 2

Pyr ligand with BH charges

molecule.res

L1l INT 0

CORRECT OMIT DU BEG

0.0000

1 DUMM DU M 0 -1 -2 0.000 .0 .0 .00000

2 DUMM DU M 1 0 -1 1.449 .0 .0 .00000

3 DUMM DU M 2 1 0 1.523 111.21 .0 .00000

4 C21 c3 M 3 2 1 1.540 111.208 -180.000 -0.056800

5 H16 hc E 4 3 2 1.095 97.979 108.218 0.047367

6 H17 hc E 4 3 2 1.099 148.231 -108.967 0.047367

7 H18 hc E 4 3 2 1.096 82.027 1.056 0.047367

8 C20 ca M 4 3 2 1.513 39.518 -137.526 -0.067300

9 C19 ca B 8 4 3 1.405 120.892 -108.897 -0.124000

10 C17 ca S 9 8 4 1.399 121.367 -178.067 -0.109000

11 H13 ha E 10 9 8 1.084 120.269 178.817 0.142500

12 H15 ha E 9 8 4 1.087 119.501 1.283 0.136500

13 C18 ca M 8 4 3 1.405 120.998 72.254 -0.124000

14 H14 ha E 13 8 4 1.086 119.503 -1.700 0.136500

15 C16 ca M 13 8 4 1.398 121.179 179.087 -0.109000

16 H12 ha E 15 13 8 1.086 120.184 178.636 0.142500

17 C5 ca M 15 13 8 1.399 119.726 -0.697 -0.024100

18 S1 ss M 17 15 13 1.853 118.710 -177.871 -0.167000

19 C4 c3 M 18 17 15 1.888 100.289 -136.344 0.163200

20 H7 h2 E 19 18 17 1.098 107.006 30.620 0.074700

21 C6 c3 B 19 18 17 1.535 109.627 149.769 0.119100

22 O4 oh S 21 19 18 1.449 110.440 -67.707 -0.606800

23 H8 ho E 22 21 19 0.979 109.786 62.366 0.428000

24 H9 h1 E 21 19 18 1.095 109.932 56.542 0.093700

25 O3 os M 19 18 17 1.442 108.094 -89.157 -0.392600

26 C2 c3 M 25 19 18 1.476 113.495 179.828 0.088100

27 C1 c3 3 26 25 19 1.523 104.453 -178.230 0.142400

28 O1 oh S 27 26 25 1.451 110.384 57.182 -0.596800

29 H1 ho E 28 27 26 0.979 107.603 -65.072 0.415000

30 H2 h1 E 27 26 25 1.093 110.268 174.118 0.067700

31 H3 h1 E 27 26 25 1.094 108.899 -66.208 0.067700

32 H6 h1 E 26 25 19 1.101 108.234 -61.156 0.055700

33 C3 c3 M 26 25 19 1.532 110.568 58.757 0.128100

34 O2 oh S 33 26 25 1.442 107.829 67.267 -0.583800

35 H4 ho E 34 33 26 0.989 107.393 178.062 0.429000

36 H5 h1 E 33 26 25 1.100 108.619 -172.502 0.068700

37 C7 c3 M 33 26 25 1.550 109.426 -54.590 0.041000

38 H10 h1 E 37 33 26 1.097 108.517 -63.009 0.078700

39 N1 na M 37 33 26 1.470 111.480 179.854 0.049400

40 N2 nc S 39 37 33 1.394 121.613 -18.629 -0.194800

41 N3 nd E 40 39 37 1.319 107.425 -179.542 -0.317000

42 C8 cc M 39 37 33 1.359 128.473 162.254 -0.155600

43 H11 h4 E 42 39 37 1.073 122.372 1.385 0.219000

44 C9 cd M 42 39 37 1.395 105.463 179.337 0.288800

45 C10 ca M 44 42 39 1.457 129.362 179.912 -0.178500

46 C15 ca B 45 44 42 1.406 124.327 166.739 0.168400

47 C14 ca S 46 45 44 1.388 122.814 179.235 0.004900

48 F3 f E 47 46 45 1.392 116.799 -177.561 -0.128400

49 F4 f E 46 45 44 1.377 120.649 -0.116 -0.109900

50 C11 ca M 45 44 42 1.404 121.979 -12.962 0.168400

51 F1 f E 50 45 44 1.390 119.173 0.628 -0.109900

52 C12 ca M 50 45 44 1.384 123.952 179.284 0.004900

53 F2 f E 52 50 45 1.393 117.159 -177.460 -0.128400

54 C13 ca M 52 50 45 1.417 122.741 1.371 0.194600

55 N4 nh M 54 52 50 1.373 123.376 179.388 -0.649000

56 C22 c3 M 55 54 52 1.491 124.159 -18.344 0.195800

57 H19 h1 E 56 55 54 1.093 110.488 -46.916 0.059200

58 H23 h1 E 56 55 54 1.095 110.944 72.748 0.059200

59 C24 c3 M 56 55 54 1.538 103.299 -168.020 -0.089400

60 H22 hc E 59 56 55 1.094 111.896 -153.565 0.055700

61 H26 hc E 59 56 55 1.097 110.357 86.126 0.055700

62 C25 c3 M 59 56 55 1.542 103.258 -31.617 -0.089400

63 H21 hc E 62 59 56 1.093 113.038 160.617 0.055700

64 H24 hc E 62 59 56 1.098 110.161 -78.570 0.055700

65 C23 c3 M 62 59 56 1.537 103.366 39.389 0.195800

66 H20 h1 E 65 62 59 1.095 111.203 87.754 0.059200

67 H25 h1 E 65 62 59 1.094 112.958 -150.794 0.059200

LOOP

C5 C17

C7 C6

C9 N3

C13 C14

C23 N4

IMPROPER

C21 C18 C20 C19

C20 C17 C19 H15

C5 C19 C17 H13

C16 C20 C18 H14

C18 C5 C16 H12

C16 C17 C5 S1

C7 C8 N1 N2

C9 H11 C8 N1

C10 C8 C9 N3

C15 C11 C10 C9

C10 C14 C15 F4

C15 C13 C14 F3

C10 C12 C11 F1

C11 C13 C12 F2

C14 C12 C13 N4

C22 C23 N4 C13

DONE

STOP

0 0 2

OH ligand with BH charges

molecule.res

L1m INT 0

CORRECT OMIT DU BEG

0.0000

1 DUMM DU M 0 -1 -2 0.000 .0 .0 .00000

2 DUMM DU M 1 0 -1 1.449 .0 .0 .00000

3 DUMM DU M 2 1 0 1.523 111.21 .0 .00000

4 F3 f M 3 2 1 1.540 111.208 -180.000 -0.111900

5 C14 ca M 4 3 2 1.390 142.446 47.535 0.036400

6 C15 ca S 5 4 3 1.388 120.645 156.958 0.159400

7 F4 f E 6 5 4 1.375 117.233 0.674 -0.103900

8 C13 ca M 5 4 3 1.394 116.927 -23.548 0.111100

9 O5 oh S 8 5 4 1.372 122.865 0.321 -0.463100

10 H19 ho E 9 8 5 0.979 110.865 -0.071 0.441000

11 C12 ca M 8 5 4 1.396 117.222 -179.492 0.036400

12 F2 f E 11 8 5 1.375 119.937 179.898 -0.111900

13 C11 ca M 11 8 5 1.388 120.099 -0.170 0.159400

14 F1 f E 13 11 8 1.387 117.099 -179.864 -0.103900

15 C10 ca M 13 11 8 1.407 123.717 -0.074 -0.150500

16 C9 cd M 15 13 11 1.459 121.420 -179.663 0.272800

17 N3 nd S 16 15 13 1.388 122.373 164.132 -0.314000

18 N2 nc E 17 16 15 1.317 109.153 -179.740 -0.190800

19 C8 cc M 16 15 13 1.395 129.468 -15.311 -0.147600

20 H11 h4 E 19 16 15 1.072 132.324 -2.808 0.221000

21 N1 na M 19 16 15 1.359 105.339 179.614 0.052400

22 C7 c3 M 21 19 16 1.472 128.475 179.489 0.039000

23 C6 c3 B 22 21 19 1.538 111.390 -72.067 0.119100

24 O4 oh S 23 22 21 1.448 105.985 60.548 -0.608800

25 H8 ho E 24 23 22 0.980 109.819 179.090 0.430000

26 H9 h1 E 23 22 21 1.096 109.460 -60.470 0.093700

27 H10 h1 E 22 21 19 1.098 106.521 44.714 0.080700

28 C3 c3 M 22 21 19 1.549 111.523 163.027 0.127100

29 O2 oh S 28 22 21 1.442 111.835 60.641 -0.582800

30 H4 ho E 29 28 22 0.988 107.548 -62.835 0.428000

31 H5 h1 E 28 22 21 1.100 108.302 -61.852 0.069700

32 C2 c3 M 28 22 21 1.533 109.329 179.909 0.088100

33 C1 c3 3 32 28 22 1.524 113.993 -172.050 0.142400

34 O1 oh S 33 32 28 1.451 110.310 178.572 -0.596800

35 H1 ho E 34 33 32 0.980 107.668 -65.272 0.415000

36 H2 h1 E 33 32 28 1.092 110.306 -64.581 0.067700

37 H3 h1 E 33 32 28 1.094 109.004 55.192 0.067700

38 H6 h1 E 32 28 22 1.101 109.470 64.693 0.056700

39 O3 os M 32 28 22 1.476 110.605 -54.636 -0.391600

40 C4 c3 M 39 32 28 1.442 113.458 58.793 0.163200

41 H7 h2 E 40 39 32 1.098 111.194 62.757 0.075700

42 S1 ss M 40 39 32 1.886 108.146 -179.930 -0.166000

43 C5 ca M 42 40 39 1.853 100.440 -89.637 -0.026100

44 C16 ca M 43 42 40 1.399 118.702 -135.929 -0.108500

45 H12 ha E 44 43 42 1.085 120.117 2.837 0.142500

46 C18 ca M 44 43 42 1.398 119.681 -177.769 -0.124000

47 H14 ha E 46 44 43 1.087 119.246 -179.869 0.137500

48 C20 ca M 46 44 43 1.405 121.220 -0.707 -0.065300

49 C21 c3 3 48 46 44 1.513 120.980 179.116 -0.057800

50 H16 hc E 49 48 46 1.095 111.469 148.525 0.047700

51 H17 hc E 49 48 46 1.099 111.155 -91.950 0.047700

52 H18 hc E 49 48 46 1.095 111.442 27.655 0.047700

53 C19 ca M 48 46 44 1.405 118.074 0.207 -0.124000

54 H15 ha E 53 48 46 1.087 119.459 -179.859 0.137500

55 C17 ca M 53 48 46 1.398 121.396 0.836 -0.108500

56 H13 ha E 55 53 48 1.084 120.272 178.712 0.142500

LOOP

C10 C15

N1 N2

C4 C6

C17 C5

IMPROPER

C15 C13 C14 F3

C10 C14 C15 F4

C12 C14 C13 O5

C11 C13 C12 F2

C12 C10 C11 F1

C11 C15 C10 C9

C10 C8 C9 N3

C9 H11 C8 N1

C7 C8 N1 N2

C16 C17 C5 S1

C5 C18 C16 H12

C16 C20 C18 H14

C21 C18 C20 C19

C20 C17 C19 H15

C5 C19 C17 H13

DONE

STOP

0 0 2

NH2 ligand with BH charges

molecule.res

L1n INT 0

CORRECT OMIT DU BEG

0.0000

1 DUMM DU M 0 -1 -2 0.000 .0 .0 .00000

2 DUMM DU M 1 0 -1 1.449 .0 .0 .00000

3 DUMM DU M 2 1 0 1.523 111.21 .0 .00000

4 F3 f M 3 2 1 1.540 111.208 -180.000 -0.124900

5 C14 ca M 4 3 2 1.390 141.042 48.339 0.009400

6 C15 ca S 5 4 3 1.388 120.121 157.340 0.170900

7 F4 f E 6 5 4 1.376 116.976 0.751 -0.107900

8 C13 ca M 5 4 3 1.403 117.282 -23.106 0.156600

9 N4 nh B 8 5 4 1.367 122.130 0.290 -0.820200

10 H19 hn E 9 8 5 1.005 120.165 179.798 0.424300

11 H20 hn E 9 8 5 1.004 120.122 0.000 0.424300

12 C12 ca M 8 5 4 1.402 115.580 -179.412 0.009400

13 F2 f E 12 8 5 1.390 117.815 179.831 -0.124900

14 C11 ca M 12 8 5 1.384 121.727 -0.315 0.170900

15 F1 f E 14 12 8 1.389 117.336 -179.805 -0.107900

16 C10 ca M 14 12 8 1.406 123.210 -0.001 -0.172500

17 C9 cd M 16 14 12 1.460 121.527 -179.597 0.282800

18 N3 nd S 17 16 14 1.387 122.464 163.204 -0.316000

19 N2 nc E 18 17 16 1.318 109.135 -179.676 -0.193800

20 C8 cc M 17 16 14 1.394 129.384 -16.085 -0.152600

21 H11 h4 E 20 17 16 1.073 132.264 -2.860 0.219000

22 N1 na M 20 17 16 1.360 105.409 179.454 0.050400

23 C7 c3 M 22 20 17 1.471 128.520 179.506 0.040000

24 C6 c3 B 23 22 20 1.538 111.475 -73.104 0.119100

25 O4 oh S 24 23 22 1.449 105.986 60.774 -0.607800

26 H8 ho E 25 24 23 0.979 109.778 179.875 0.429000

27 H9 h1 E 24 23 22 1.096 109.453 -60.243 0.093700

28 H10 h1 E 23 22 20 1.097 106.562 43.715 0.079700

29 C3 c3 M 23 22 20 1.549 111.475 162.019 0.127100

30 O2 oh S 29 23 22 1.441 111.865 60.578 -0.582800

31 H4 ho E 30 29 23 0.989 107.456 -61.844 0.429000

32 H5 h1 E 29 23 22 1.101 108.223 -61.870 0.068700

33 C2 c3 M 29 23 22 1.532 109.402 179.949 0.088100

34 C1 c3 3 33 29 23 1.525 114.011 -172.052 0.142400

35 O1 oh S 34 33 29 1.451 110.282 178.363 -0.596800

36 H1 ho E 35 34 33 0.979 107.670 -65.073 0.415000

37 H2 h1 E 34 33 29 1.092 110.285 -64.729 0.067700

38 H3 h1 E 34 33 29 1.093 108.930 55.000 0.067700

39 H6 h1 E 33 29 23 1.101 109.534 64.626 0.055700

40 O3 os M 33 29 23 1.475 110.610 -54.685 -0.391600

41 C4 c3 M 40 33 29 1.443 113.468 58.734 0.163200

42 H7 h2 E 41 40 33 1.098 111.158 62.863 0.074700

43 S1 ss M 41 40 33 1.888 108.111 -179.862 -0.166000

44 C5 ca M 43 41 40 1.853 100.448 -89.392 -0.025100

45 C16 ca M 44 43 41 1.398 118.692 -136.260 -0.109000

46 H12 ha E 45 44 43 1.086 120.120 2.858 0.142500

47 C18 ca M 45 44 43 1.398 119.762 -177.731 -0.124000

48 H14 ha E 47 45 44 1.085 119.309 -179.886 0.137500

49 C20 ca M 47 45 44 1.405 121.133 -0.779 -0.066300

50 C21 c3 3 49 47 45 1.512 120.960 179.090 -0.056800

51 H16 hc E 50 49 47 1.095 111.506 148.765 0.047700

52 H17 hc E 50 49 47 1.099 111.226 -91.636 0.047700

53 H18 hc E 50 49 47 1.096 111.480 28.016 0.047700

54 C19 ca M 49 47 45 1.405 118.143 0.282 -0.124000

55 H15 ha E 54 49 47 1.086 119.533 -179.904 0.137500

56 C17 ca M 54 49 47 1.398 121.345 0.788 -0.109000

57 H13 ha E 56 54 49 1.085 120.272 178.798 0.142500

LOOP

C10 C15

N1 N2

C4 C6

C17 C5

IMPROPER

C15 C13 C14 F3

C10 C14 C15 F4

C12 C14 C13 N4

C13 H20 N4 H19

C11 C13 C12 F2

C12 C10 C11 F1

C11 C15 C10 C9

C10 C8 C9 N3

C9 H11 C8 N1

C7 C8 N1 N2

C16 C17 C5 S1

C5 C18 C16 H12

C16 C20 C18 H14

C21 C18 C20 C19

C20 C17 C19 H15

C5 C19 C17 H13

DONE

STOP
